# Supplementary material for: Tackling recalcitrant Pseudomonas aeruginosa infections in critical illness via anti-virulence monotherapy
Source: Nat Commun. 2022 Aug 30;13:5103. doi: 10.1038/s41467-022-32833-9 (PMC9428149; doi:10.1038/s41467-022-32833-9)
Supplement: Supplementary file 1 — Supplementary Information [file 41467_2022_32833_MOESM1_ESM.pdf]

Supplementary Information

Tackling Recalcitrant *Pseudomonas aeruginosa* Infections In Critical Illness via Anti-virulence Monotherapy

Vijay K. Singh[1,2,3], Marianna Almpani[1,2,3], Damien Maura[1,2,3,11], Tomoe Kitao[1,2,3,11], Livia Ferrari[4], Stefano Fontana[5], Gabriella Bergamini[4], Elisa Calcaterra[4], Chiara Pignaffo[5], Michele Negri[6], Thays de Oliveira Pereira[7], Frances Skinner[8], Manos Gkikas[8], Danielle Andreotti[9], Antonio Felici[10,11], Eric Déziel[7], Francois Lépine[7], Laurence G. Rahme[1,2,3*]

[1]Department of Surgery, Harvard Medical School and Massachusetts General Hospital, Boston, USA

[2]Shriners Hospitals for Children, Boston, USA

[3]Department of Microbiology, Harvard Medical School, Boston, MA 02115, USA

[4]Translational Biology Department, Aptuit (Verona) S.rl, *an Evotec Company*, 37135 Via A. Fleming 4, Verona, Italy

[5]DMPK Department, Aptuit (Verona) S.rl, *an Evotec Company*, 37135 Via A. Fleming 4, Verona, Italy

[6]*In vitro* Chemotherapy Laboratory, Aptuit (Verona) S.r.l., *an Evotec Company*, 37135 Via A. Fleming 4, Verona, Italy

[7]Centre Armand-Frappier Santé Biotechnologie, Institut National de la Recherche Scientifique (INRS), Laval, Quebec, H7V 1B7 Canada

[8]Department of Chemistry, University of Massachusetts Lowell, Lowell, MA 01854, USA

[9]Global Synthetic Chemistry Department, Aptuit (Verona) S.r.l., *an Evotec Company*, 37135 Via A. Fleming 4, Verona, Italy

[10]Department of Microbiology Discovery, *In Vitro* Biology, Aptuit (Verona) S.r.l., *an Evotec Company*, 37135 Via A. Fleming 4, Verona, Italy

[11]Current address:

D. Maura, Voyager Therapeutics, Cambridge, MA 02139, USA

T. Kitao, Department of Microbiology, Graduate School of Medicine, Gifu University, Gifu 501-1194, Japan

A Felici, Academic Partnership, Evotec SE, 37135 Via A. Fleming 4, Verona, Italy

[*]**Corresponding author:**

e-mail: rahme@molbio.mgh.harvard.edu (LGR)

address: Massachusetts General Hospital, 340 Their Research Building, 50 Blossom Street, Boston, MA 02114; phone: 617-724-5003

These authors contributed equally: Vijay K. Singh, Marianna Almpani, Damien Maura

Structure activity relationship (SAR) studies.

The assessment of inhibitors potency was based on pyocyanin production, *pqs* operon gene expression using the *pqsA*-GFP reporter, and the production of the MvfR-regulated low molecular weight molecules HHQ, PQS, HQNO, DHQ, 2-AA in the presence of 50μM of each inhibitor. Assessment of the anthranilic acid (AA) production was used as additional control of the inhibition of the low molecular weight molecules since their synthesis depends on AA, their primary precursor.

Supplementary Table 1. Optimization starting with M17

SAR studies of N 2-[(4-fluoro-2-methylphenyl)amino]-N-phenylacetamide as anti-MvfR agents via exploration of the aryl portion of the N-phenylacetamide side.

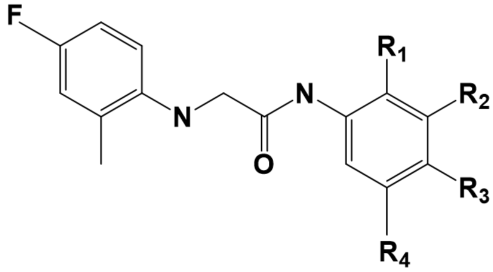

Compound					% Production							
	R_1	R_2	R_3	R_4	Pyocyanin	*pqsA*-GFP	HHQ	PQS	HQNO	2-AA	DHQ	AA
M17			CN		67	32	3	11	17	101	135	100
G1		CN			103	84	100	109	101	122	-	-
G2			CF_3		61	46	110	93	86	68	81	-
G3	CN				113	96	99	122	111	123	-	-
G4			F		84	92	95	88	92	80	85	-
D2	Br				101	96	-	-	-	-	-	-
D5			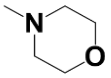		102	100	-	-	-	-	-	-
D7	Me		Br		89	70	-	-	-	-	-	-
D9			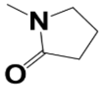		107	105	-	-	-	-	-	-
D10	F			F	90	89	-	-	-	-	-	-
D12	Br		CH_3		108	92	-	-	-	-	-	-
D13		CN			100	90	-	-	-	-	-	-
D14		NO_2			85	77	-	-	-	-	-	-
D15			OMe		102	87	-	-	-	-	-	-
D19			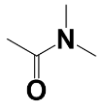		109	100	-	-	-	-	-	-
D21	Me		Me		113	87	-	-	-	-	-	-
D22		Cl			96	71	-	-	-	-	-	-
D30	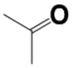				91	94	-	-	-	-	-	-
D31		CH_3	Br		69	47	-	-	-	-	-	-
D32		OMe			92	94	-	-	-	-	-	-

Supplementary Table 2. SAR studies of N-(4-cyanophenyl)-2-(phenylamino) acetamide as anti-MvfR agents via exploration of the aryl portion of the 2-phenylamino portion of the molecule.

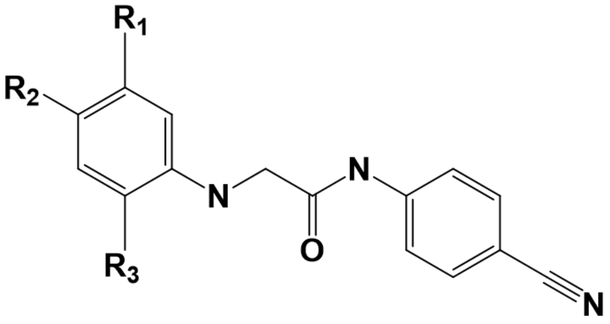

Compound				% Production							
	R_1	R_2	R_3	Pyocyanin	pqsA-GFP	HHQ	PQS	HQNO	2-AA	DHQ	AA
G8		F		86	63	87	124	108	112	-	-
G9			Me	94	93	76	124	92	64	59	-
D3			Et	97	92	-	-	-	-	-	-
D6	F			91	68	-	-	-	-	-	-
D16		Cl		43	18	-	-	-	-	-	-
D18	F		Me	69	28	-	-	-	-	-	-
D25			F	97	84	-	-	-	-	-	-
D28	F	CH_3		60	42	-	-	-	-	-	-
D33		$CH(CH_3)_2$		59	53	-	-	-	-	-	-

Supplementary Table 3. SAR studies retaining the 4-cyanophenyl and exploring the central linker and the effect of the substituent on the second Aryl group.

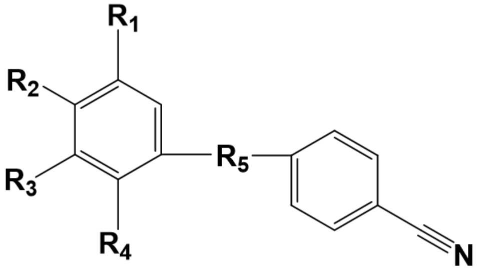

Compound				% Production							
	R_2	R_4	R_5	Pyocyanin	*pqsA*-GFP	HHQ	PQS	HQNO	2-AA	DHQ	AA
G5	F	Me	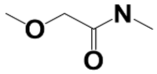	98	98	114	114	111	63	83	54
G6	F	Me	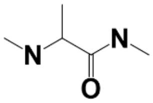	110	87	106	115	131	152	-	-
D38	F	Me	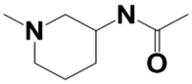	108	90	98	96	100	101	84	54
D39	F	Me	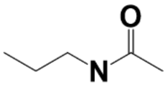	110	91	96	93	102	106	96	68
D40	F	Me	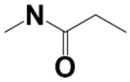	115	96	102	100	109	113	85	81
D24	F		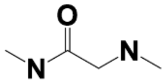	59	26	-	-	-	-	-	-
D34	F		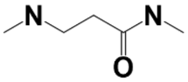	91	80	97	97	96	113	100	101
D37	F		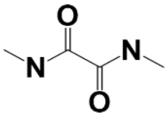	106	97	98	110	99	103	105	90
D36	F		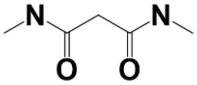	16	10	57	47	59	37	68	187
D44	F		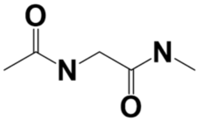	94	71	94	92	94	92	97	32
D47	F		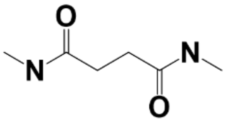	99	78	95	94	93	100	100	42
D83	CN		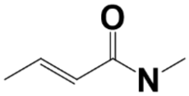	114	-	-	-	-	-	-	-

D76	F	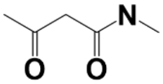	77	-	-	-	-	-	-	-
D84	F	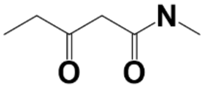	102	-	-	-	-	-	-	-
D86	F	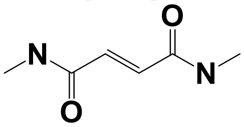	88	-	-	-	-	-	-	-
D89	F	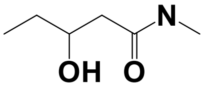	95	-	-	-	-	-	-	-
D93	C_6H_6<br>O	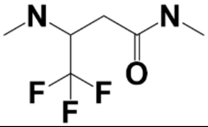	91	88	-	-	-	-	-	-

Supplementary Table 4. SAR studies exploring one side of the N'-(4-cyanophenyl)-N-aryl Malonamide

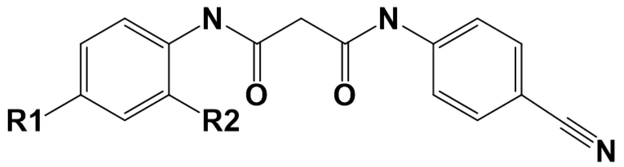

Compound			% Production							
Name	R_1	R_2	Pyocyanin	*pqsA*-GFP	HHQ	PQS	HQNO	2-AA	DHQ	AA
D41	Cl		6	8	2	9	15	6	11	739
D42	Br		6	8	2	8	14	6	11	701
D43	CN		8	8	22	24	39	19	34	531
D48	F	Me	60	44	96	90	94	77	91	59
D49	COMe		66	28	120	119	101	90	106	131
D50	$COCF_3$		84	102	107	104	103	140	89	176
D51	NO_2		3	10	3	6	18	7	12	263
D52	NH_2		87	90	92	86	92	101	103	115
D53	NHCOMe		103	85	75	88	85	93	98	56
D54	$NHCOCF_3$		94	85	103	101	101	117	108	82
D55	$NHSO_2Me$		100	131	130	115	98	95	80	117
D56	I		13	6	63	62	69	72	136	180
D69	CF_3		4	2	6	16	40	15	13	920
D57	C_6H_5O		6	4	58	36	54	38	65	298

Supplementary Table 5. SAR studies retaining the 4-phenoxy phenyl motif of the NAMs and exploring the effect of the substituents on the second Aryl group

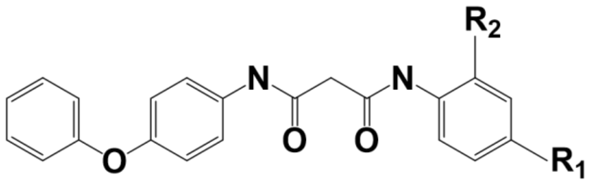

Compound			% Production							
Name	R_1	R_2	Pyocyanin	*pqsA-GFP*	HHQ	PQS	HQNO	2-AA	DHQ	AA
D57	CN		6	4	58	36	54	38	65	298
D58	NO2		6	9	20	19	23	22	45	101
D59	I		72	81	99	93	97	92	93	131
D60	NO2	NH2	6	-	-	-	-	-	-	-
D61	NO2	OH	6	7	1	6	8	12	8	112
D62	CN	NH2	7	6	2	9	13	10	16	107
D63	CN	OH	6	7	0	4	4	5	4	68
D64	Br		48	61	88	100	88	78	85	50
D67	Cl		26	41	101	106	105	84	105	155
D68	F		33	52	121	137	121	84	109	127

Supplementary Table 6. Further investigation of NAMs

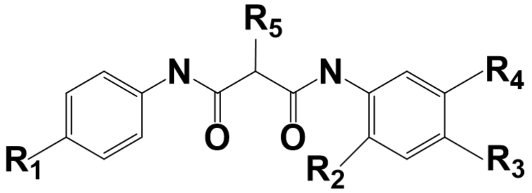

Compound						% Production							
Name	R1	R_2	R_3	R_4	R_5	Pyocyanin	*pqsA*-GFP	HHQ	PQS	HQNO	2-AA	DHQ	AA
D92	C_6H_6O	NH_2	CN			4	1	-	-	-	-	-	-
D98	C_6H_6O		CF_3			42	29	-	-	-	-	-	-
D70	CN		Me			50	-	-	-	-	-	-	-
D71	CN			Cl		13	10	106	105	127	57	92	207
D72	CN			F		30	-	-	-	-	-	-	-
D75	CN			CN		49	-	-	-	-	-	-	-
D95	CN		CN	Cl		4	6	5	10	30	11	13	774
D80	CN		CN		Me	7	10	8	20	64	16	20	832
D88	C_6H_6O		CN		F	6	2	1	4	14	7	7	832
D96	CN		CN		F	34	42	-	-	-	-	-	-
D100	C_6H_6O		CF_3		F	7	2	-	-	-	-	-	-
D94	CN		CN		Me, F	52	85	-	-	-	-	-	-
D97	CN		CN		F, F	94	95	-	-	-	-	-	-

Supplementary Table 7. NAMs analogs bearing Pyridine motif replacing one or two Aryl groups

Compound		% Production							
Name		Pyocyanin	*pqsA*-GFP	HHQ	PQS	HQNO	2-AA	DHQ	AA
D77	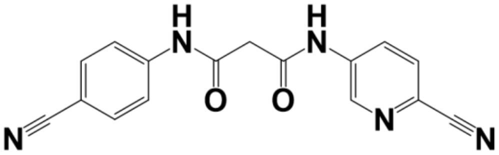	7	1	10	24	64	19	20	777
D78	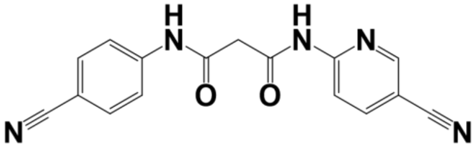	14	-	-	-	-	-	-	-
D91	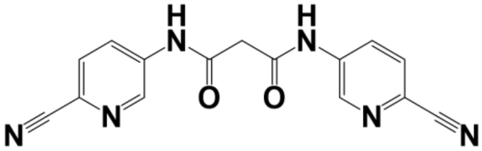	43	39	-	-	-	-	-	-

Supplementary Table 8. **Solubility assessment of NAMs in PBS.** Solubility of the 10mM compounds dissolved in DMSO was tested in the isotonic phosphate buffer (iPBS) at pH 7.4 using High-Performance Liquid Chromatography (HPLC). The solubility of each compound is expressed by the ratio of compound amount in the sample test solution to the amount of compound in the standard solution.

Compound	Solubility (μM) in PBS
D41	13
D42	15
D43	11
D57	7
D69	21
D77	39
D80	29
D88	490
D95	17
D100	5

Supplementary Table 9. Antibiotic-resistance profile of *P. aeruginosa* clinical isolates

	Antibiotic Resistance Profile							
**Strain No.**	**Amik.**	**Gent.**	**Mero.**	**Pip.**	**Tobra.**	**Cefe.**	**Aze.**	**Cip.**
LGR-4325	R	R	R	R	S	R	R	R
LGR-4326	R	R	R	S	S	S	S	R
LGR-4327	R	R	R	R	I	R	R	R
LGR-4328	S	S	S	S	S	S	S	S
LGR-4330	S	S	R	S	S	S	S	R
LGR-4331	S	S	S	S	S	S	S	S
LGR-4333	R	R	R	R	R	R	R	R
LGR-4334	R	R	S	R	R	S	S	S
LGR-4340	S	S	S	S	S	S	S	S
LGR-4343	R	R	R	R	R	R	S	S
LGR-4344	R	R	I	R	R	R	R	S
LGR-4348	S	R	R	R	R	I	S	R
LGR-4356	R	R	R	R	R	R	R	R
LGR-4362	R	R	R	R	R	R	R	S
LGR-4363	R	R	I	R	R	R	I	S
LGR-4364	R	R	R	R	R	R	I	S
LGR-4366	S	R	R	R	R	R	R	R
LGR-4368	S	S	S	S	S	S	S	S

Amik. = Amikacin, Gent. = Gentamycin, Mero. = Meropenem, Pip. = Piperacin, Tobra. = Tobramycin, Cefe. = Cefepime, Aze. = Azetromycin, Cip. = Ciprofloxacin.

R = Resistant; I = intermediate; S = sensitive.

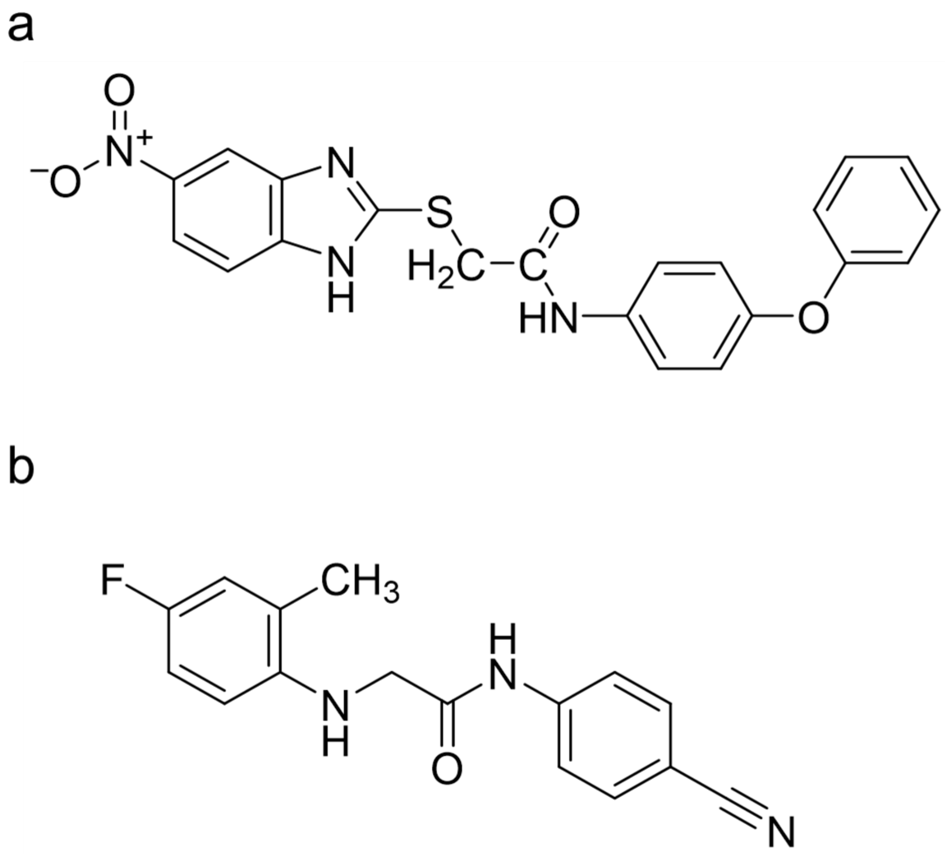

Supplementary Figure 1. Chemical structure of (a) M64 and (b) M17

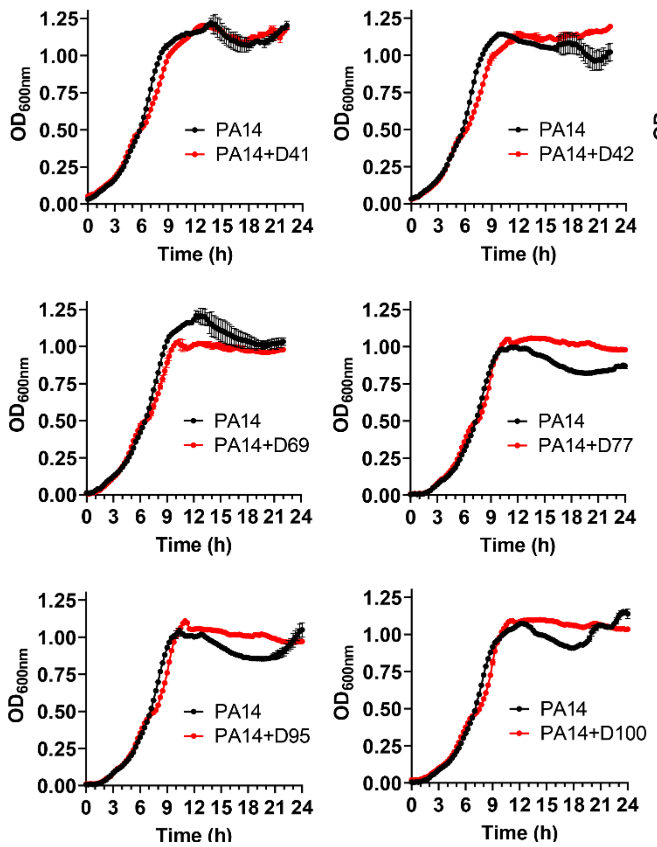

Supplementary Figure 2: NAMs do not affect the growth of the *P. aeruginosa* PA14. The growth curve of PA14 was determined with or without a compound. Cells were grown in 96-well microtiter plates in Tecan F200 microplate reader at 37˚C for 24h in the presence of 50 µM of the compounds (red) or vehicle (black), and optical density (OD) was measured at 600_{nm} every 15minute up to 24hr. Data represent n=2-3, The error bars denote ± SEM. Source data having exact replicate are provided in a Source Data file.

a

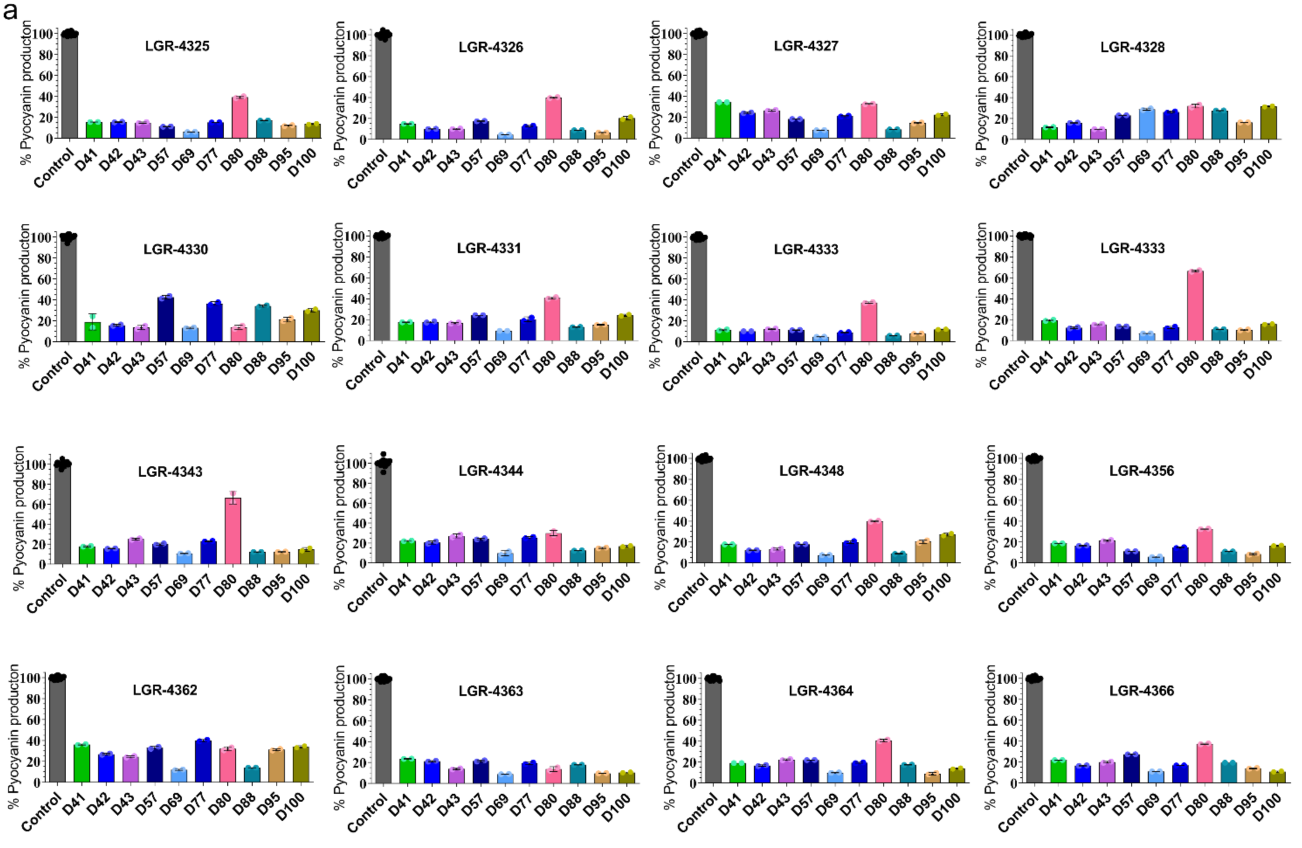

LGR-4325
LGR-4326
LGR-4327
LGR-4328
LGR-4330
LGR-4331
LGR-4333
LGR-4333
LGR-4343
LGR-4344
LGR-4348
LGR-4356
LGR-4362
LGR-4363
LGR-4364
LGR-4366
% Pyocyanin producton
0
20
40
60
80
100
Control
D41
D42
D43
D57
D69
D77
D80
D88
D95
D100

b

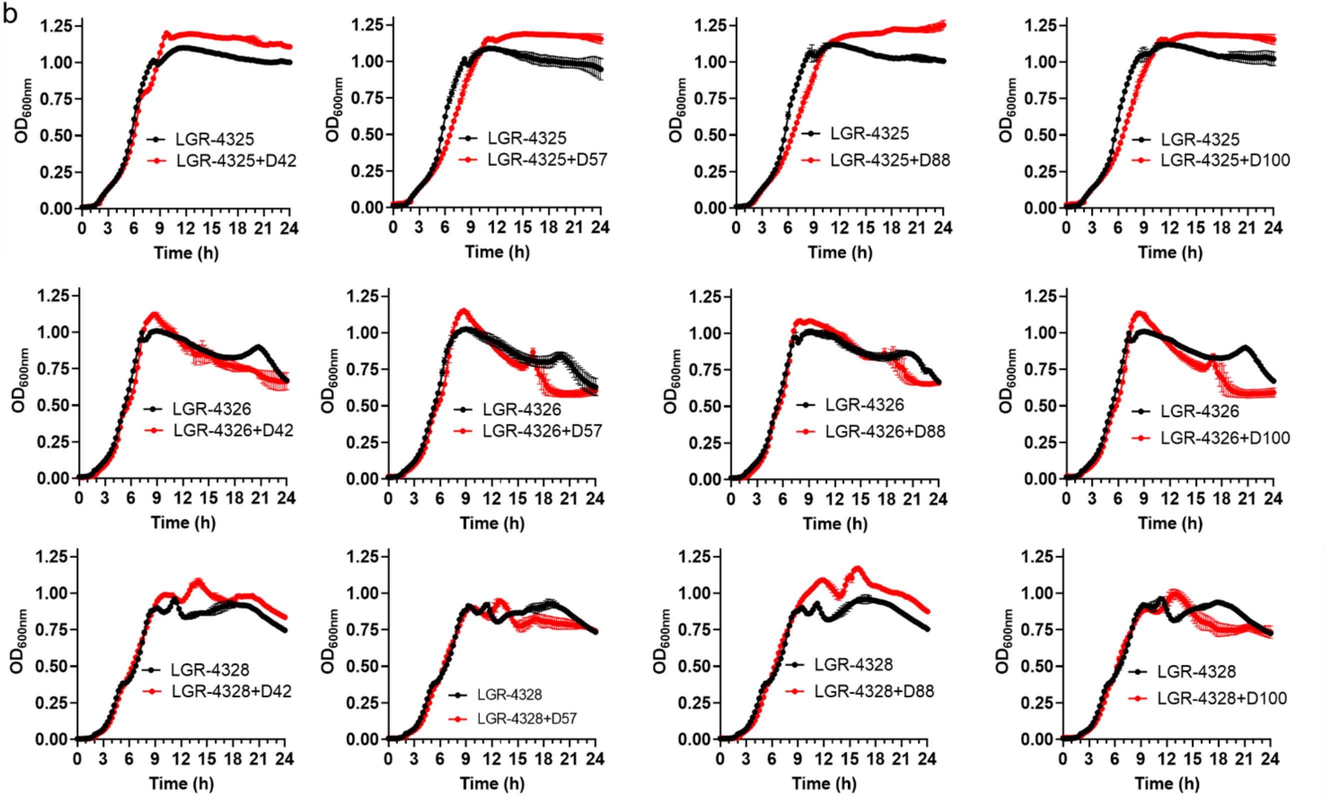

LGR-4325
LGR-4325+D42
LGR-4325+D57
LGR-4325+D88
LGR-4325+D100
LGR-4326
LGR-4326+D42
LGR-4326+D57
LGR-4326+D88
LGR-4326+D100
LGR-4328
LGR-4328+D42
LGR-4328+D57
LGR-4328+D88
LGR-4328+D100
OD600nm
0.00
0.25
0.50
0.75
1.00
1.25
Time (h)
0
3
6
9
12
15
18
21
24

Supplementary Figure 3. NAMs are highly efficacious against pyocyanin production in multi-drug resistant clinical strains of *P. aeruginosa*. a. Pyocyanin production was measured in 16 *P. aeruginosa* clinical isolates 18hr post-growth in the presence or absence of 10 µM of each NAM compound. The percent of pyocyanin production was calculated by comparing the same strain grown in the presence of the vehicle control. **b.** Representative growth curves in the presence or absence of 50µM of NAM compounds. Cells were grown in 96-well microtiter plates in Tecan F200 microplate reader at 37˚C for 24h in the presence of 50 µM of the compounds (red) or vehicle (black), and optical density (OD) was measured at 600_{nm} every 15minute up to 24hr. **a-b** Data represent n=2-3, the error bars denote ± SEM. Source data having exact replicate are provided in a Source Data file.

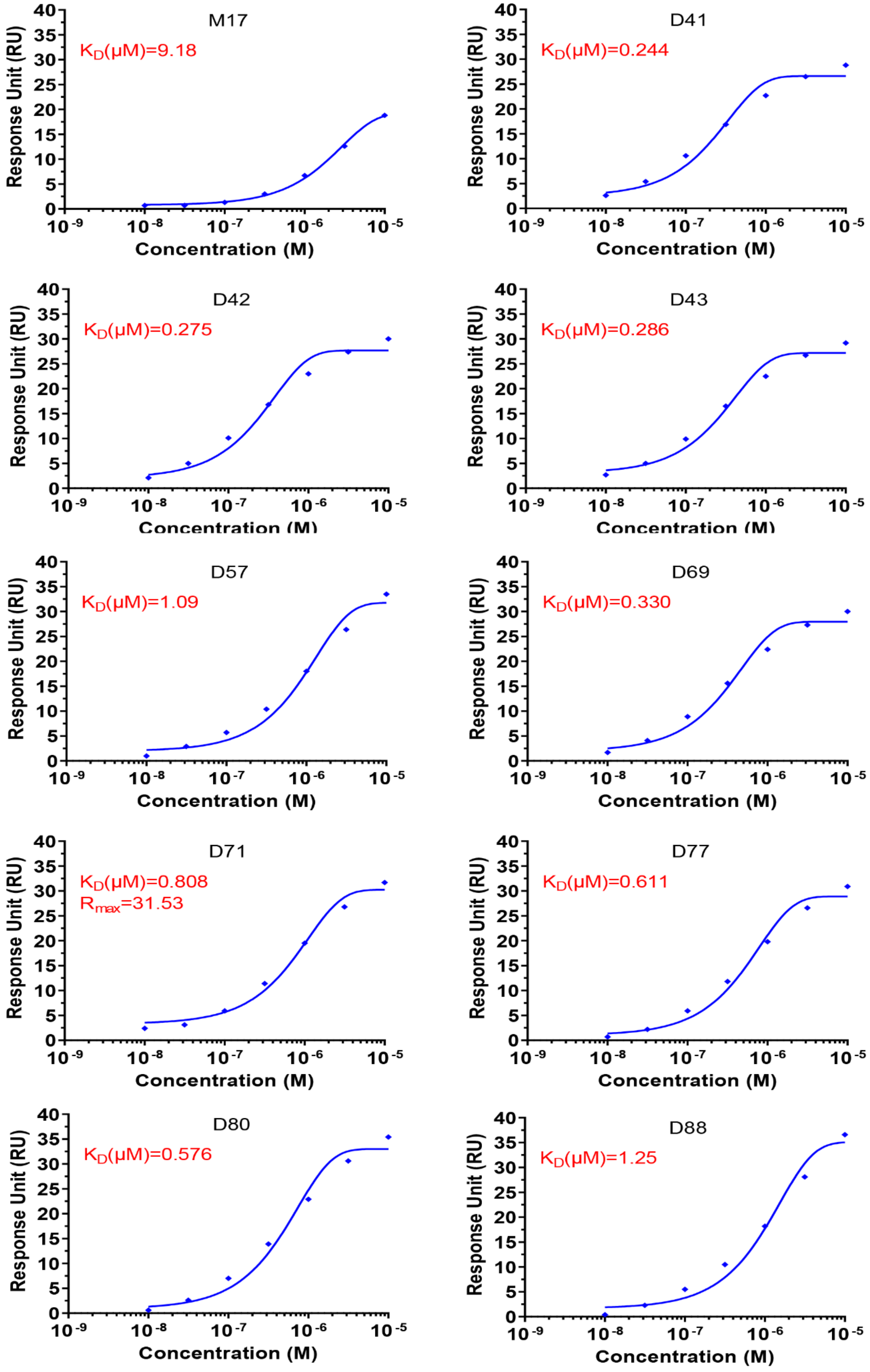

Supplementary Figure 4. Binding kinetics profile of NAMs to the MvfR protein. The binding of the compounds was assessed using Surface Plasmon Resonance (SPR).

MvfR-D88 docking result

Form#	Score (Binding energy) [kcal/mol]
1	-10.857
2	-9.605
3	-9.494
4	-9.239

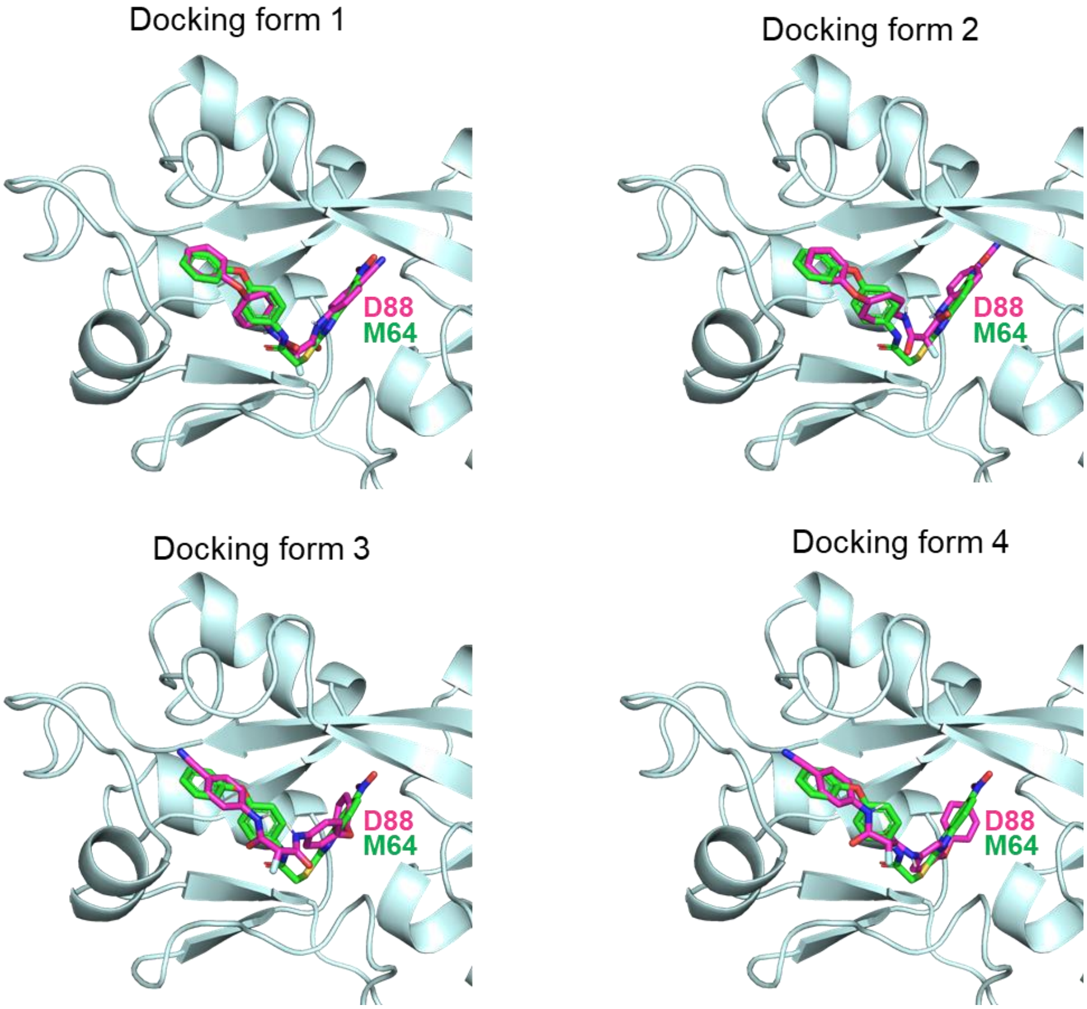

Supplementary Figure 5. Results of MvfR-D88 docking analysis using AutoDock Vina. Docking forms of D88 were superimposed with the crystal structure of MvfR-LBD in a complex with M64 (PDB ID: 6B8A). D88 and M64 were indicated with magenta and green sticks, respectively.

MvfR-M64 docking result

Form #	Score (Binding energy) [kcal/mol]
1	-9.858
2	-9.829
3	-9.304
4	-9.275

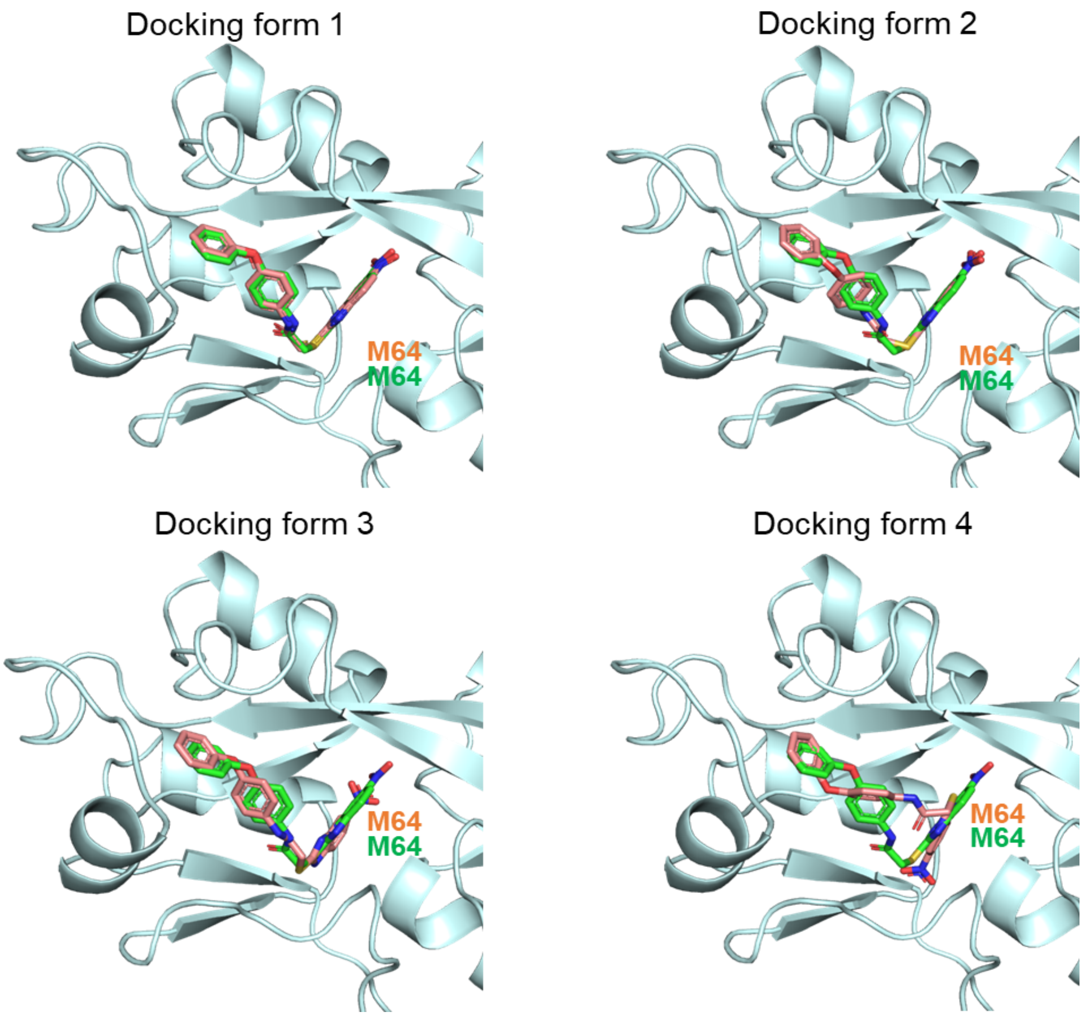

Supplementary Figure 6. Results of cross-docking analysis using M64. M64 was used for cross-docking validation. Docking forms of M64 were superimposed with the crystal structure of MvfR-LBD in a complex with M64 (PDB ID: 6B8A). Docked M64 and M64 observed in the crystal structure were indicated with orange and green sticks, respectively. In the docking form 1 with the highest score, M64 was docked at approximately the same position as M64 in the crystal structure.

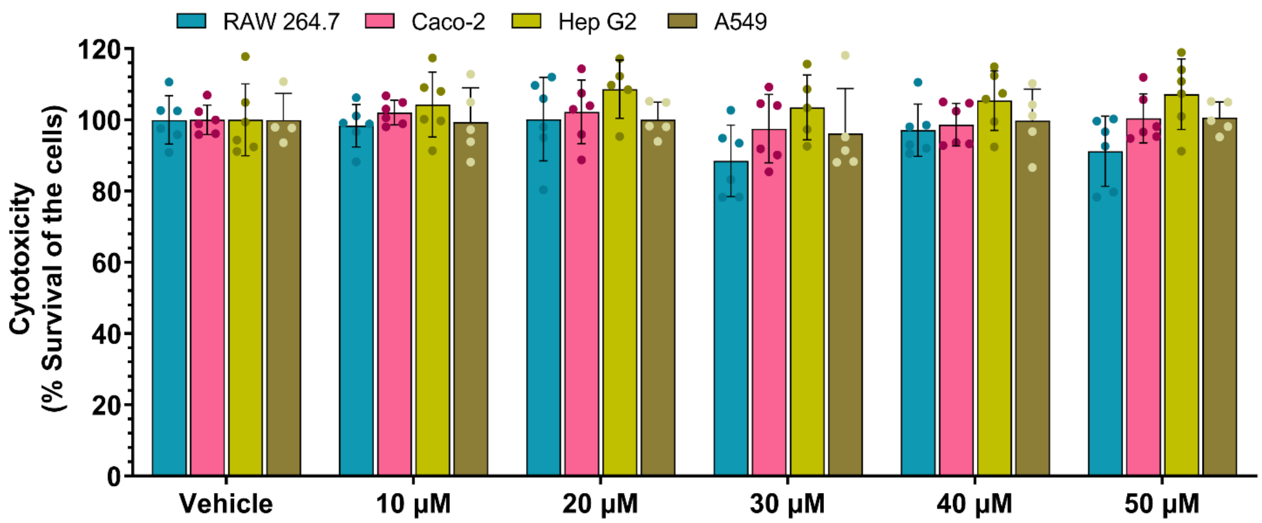

Supplementary Figure 7. Cytotoxicity assessments of the compound D88. Cell viability was assessed in four different cell types, RAW 264.7 (macrophage), Caco-2 (colon epithelial cells), Hep G2 (liver cells), and A549 (lung epithelial cells), the presence and absence of D88 at different compound concentrations (10, 20, 30, 40, and 50μM). None of the used compound concentrations exerted any cytotoxic effect in the tested cell lines. The cells' percent survival was calculated compared to the same cell type grown in the presence of vehicle control. Data represent at least n=4, each dot on the graph represents one biological replicate. The number of biological replicates used for each cell line and D88 concentration is depicted on the bar. The error bars denote ± SEM. Source data having exact n replicate is provided in a Source Data file.

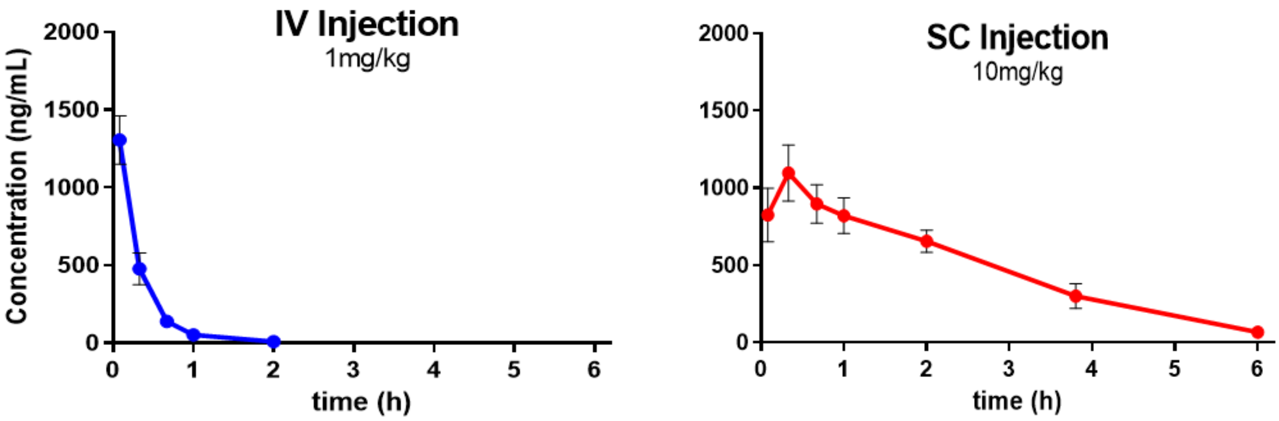

Matrix	$T_{½}$ (h)	T_{max} (h)	T_{last} (h)	C_{max} (ng/mL)	C_{last} (ng/mL)	AUC_{0-t} (h*ng/mL)
**IV**	0.27	0.080	2	1086	14.2	494
**SC**	1.25	0.33	6	1096	66.6	2892

Supplementary Figure 8. Pharmacokinetics of D88 *in vivo*. D88 was administered via intratracheal instillation 10.2 mg/Kg) in homogeneous suspension (0.5 % HPMC in water and assessed at different time points up to 6h. Data represent mean value of n=4 biological replicates, and the error bars denote ± SEM. Source data having exact replicate are provided in a Source Data file.

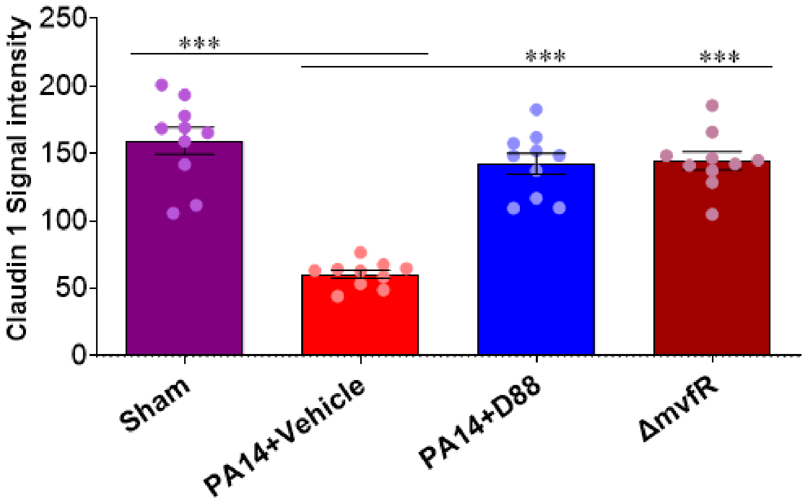

Supplementary Figure 9. Quantification of the respective confocal images. The signal intensity of claudin 1 in the PA14 infected + vehicle control group was compared to the groups of infected and D88-treated, Δ*mvfR*-infected, and sham (vehicle-treated only). Each dot represents signal intensity at different area of the image 7a obtained from n=1 for each group. Images obtained from the additional biological replicates n=2 had similar results. The error bars denote ± SEM. One-way ANOVA followed by Tukey post-test was applied. *** indicate significant differences compared to the no treatment control at P < 0.001.

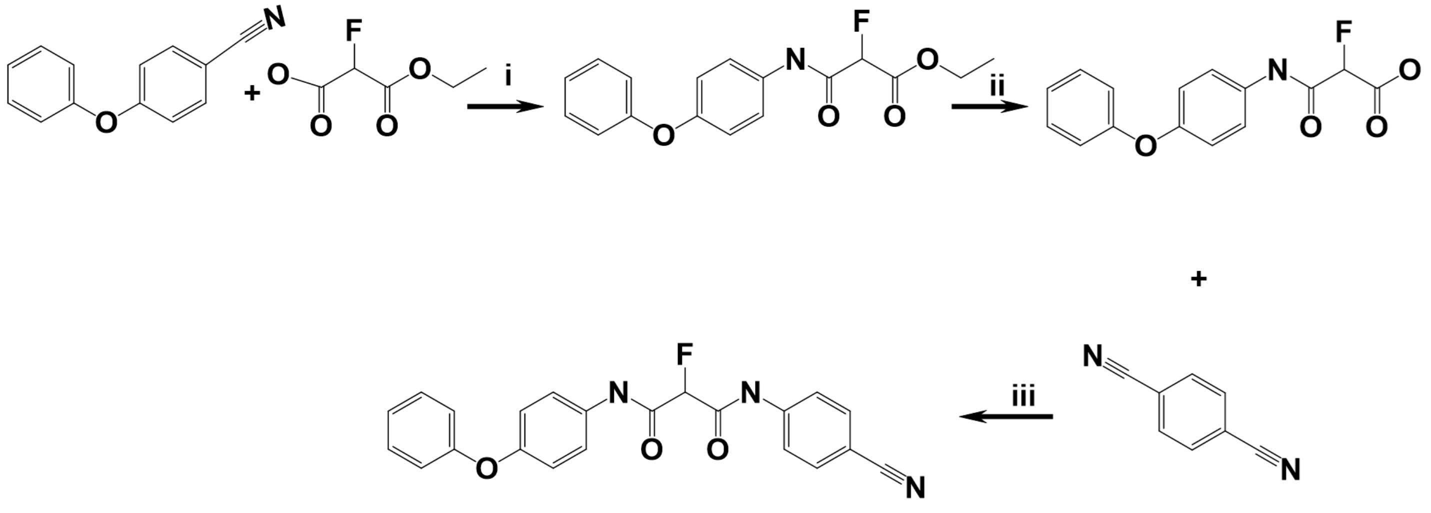

D88

Supplementary Figure 10. Route of synthesis for compound D88.

Supplementary Figure 11. Analytical data of non-commercially available compounds. a-h ^{1}H NMR and Mass spectrometry spectra shown for compounds, **a.** D47, **b.** D56, **c.** D59, **d.** D61, **e.** D62**, f.** D63, **g.** D64, **h.** D88. DMSO was used as a solvent. All compounds were synthesized by Enamine Ltd., Kyiv, Ukraine. Certificate of analysis reports compounds' purity ~ 95%.

a1

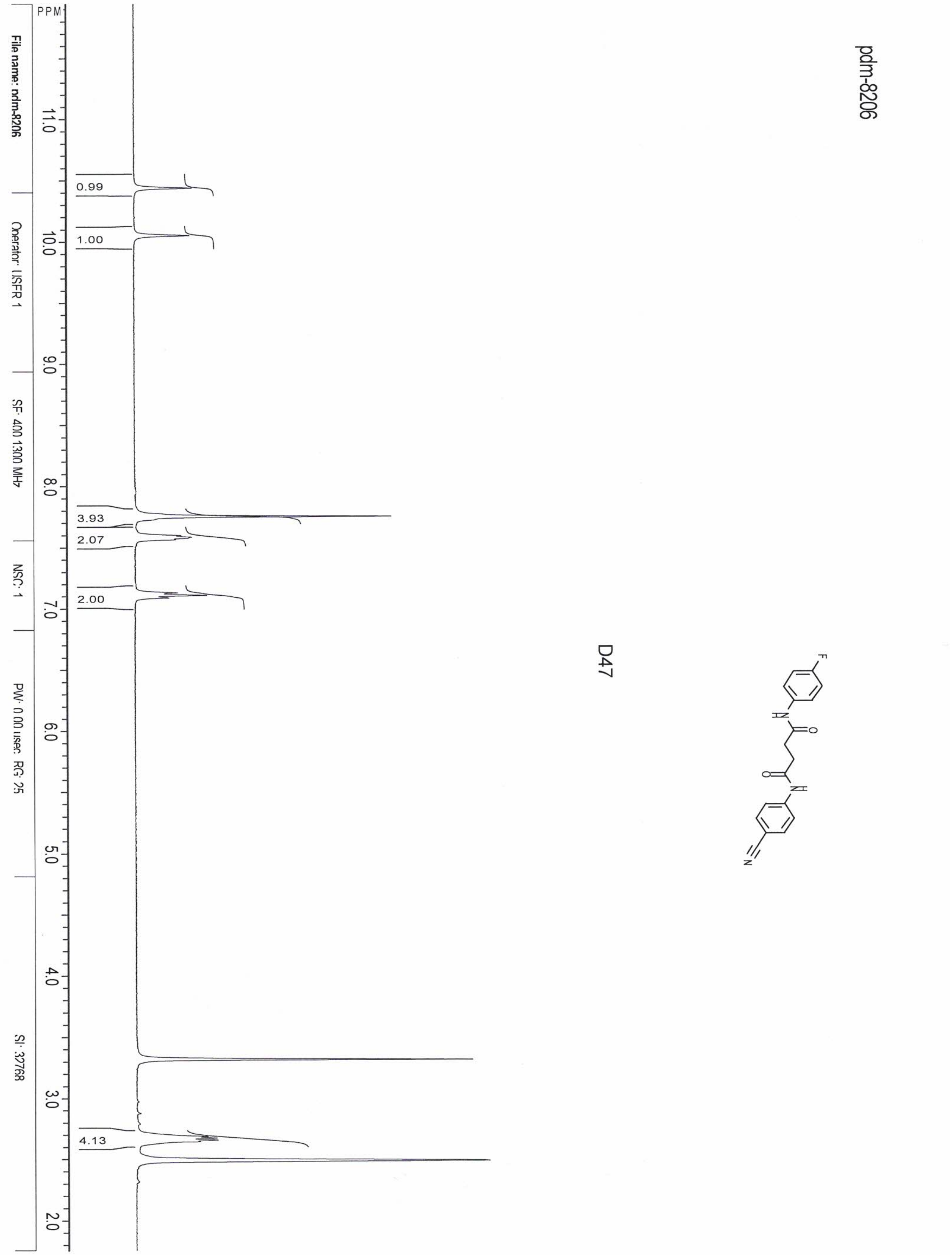

a2

axPeak: 100.00%
et_Time: 1.117 min

PDM-8206

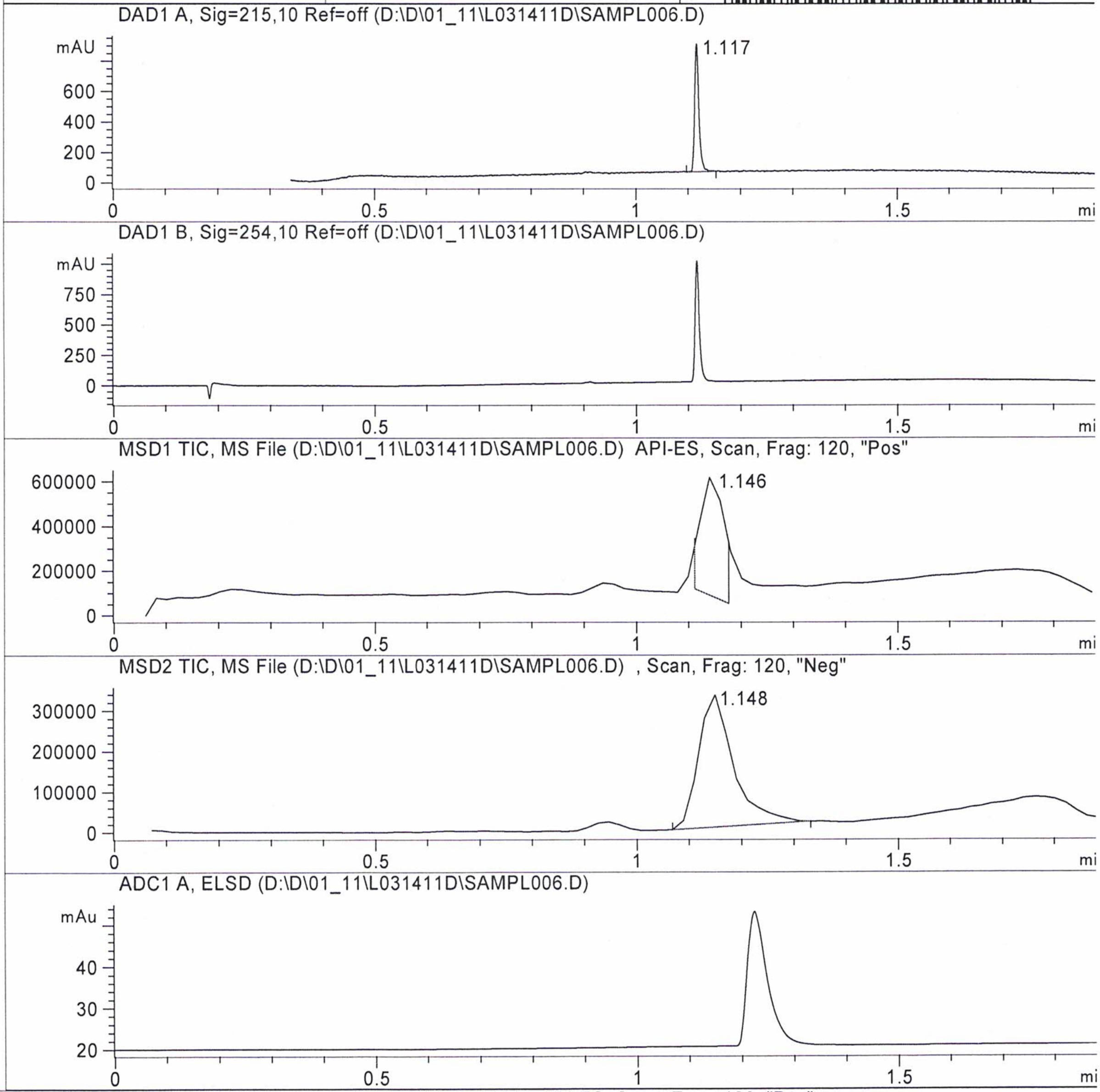

Mol Wt 0

Exact Mass

Time	Area%
1.117	100.00

RT 1.146

RT 1.148

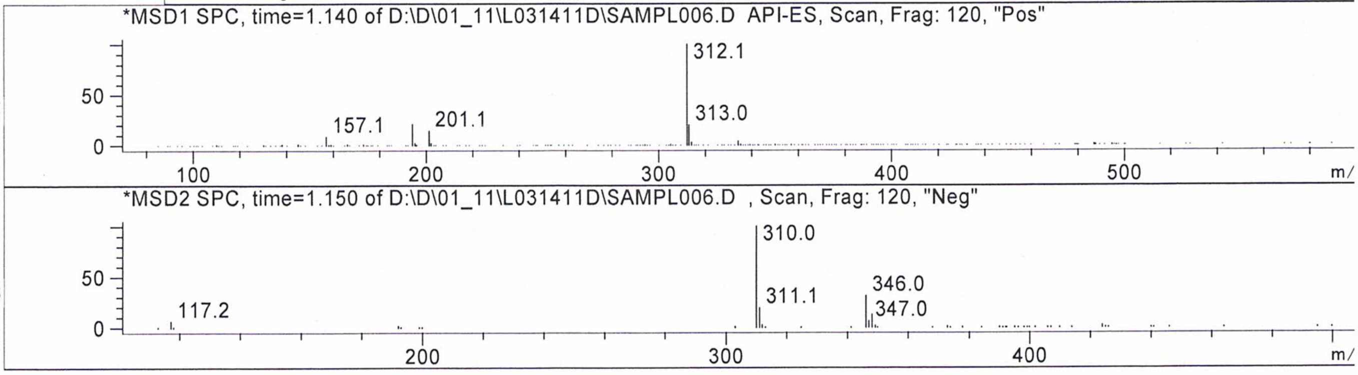

b1

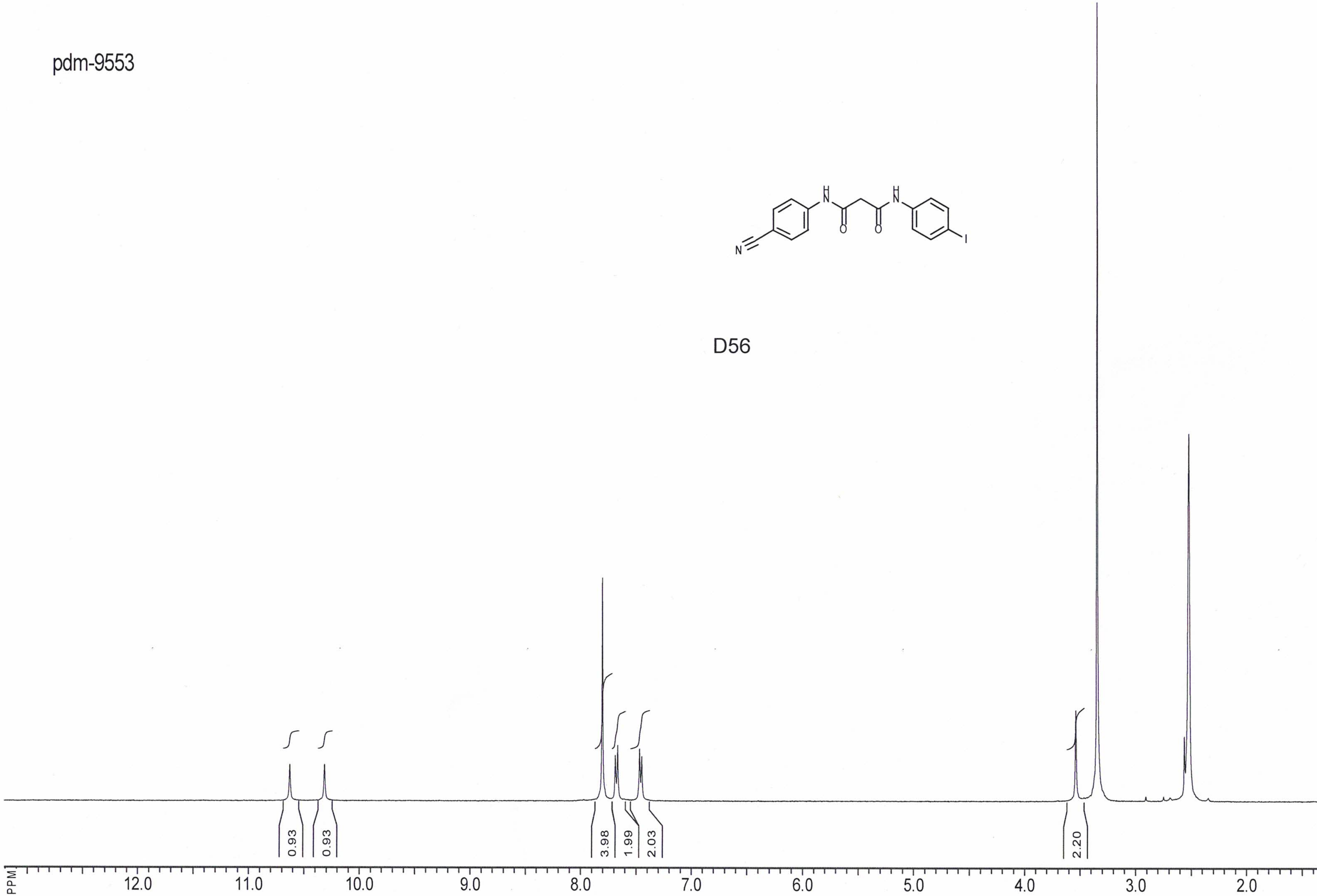

**File name: pdm-9553**	Operator: USER 1	SF: 400.1300 MHz	NSC: 1	PW: 0.00 usec, RG: 25	SI: 32
Date: 08-Aug-2017	Solvent: DMSO	SW: 8224 Hz	TE: 298 K	AQ: 1.99 sec, RD: 0.00 sec	

b2

MaxPeak: 100.00%
Ret_Time: 1.385 min

PDM-9553

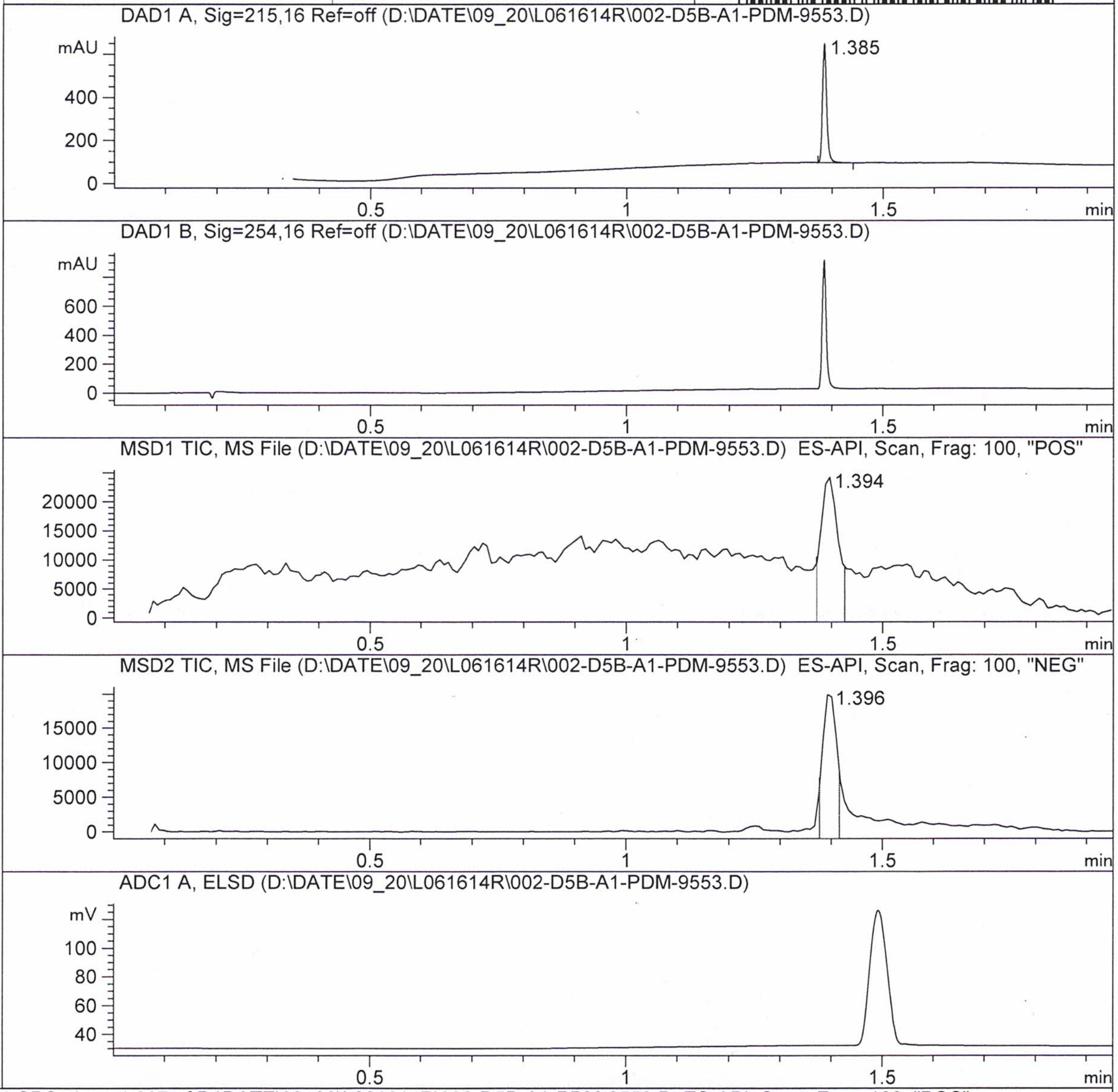

Mol Wt 0

Exact Mass

Time	Area%
1.385	100.00

RT 1.394

RT 1.396

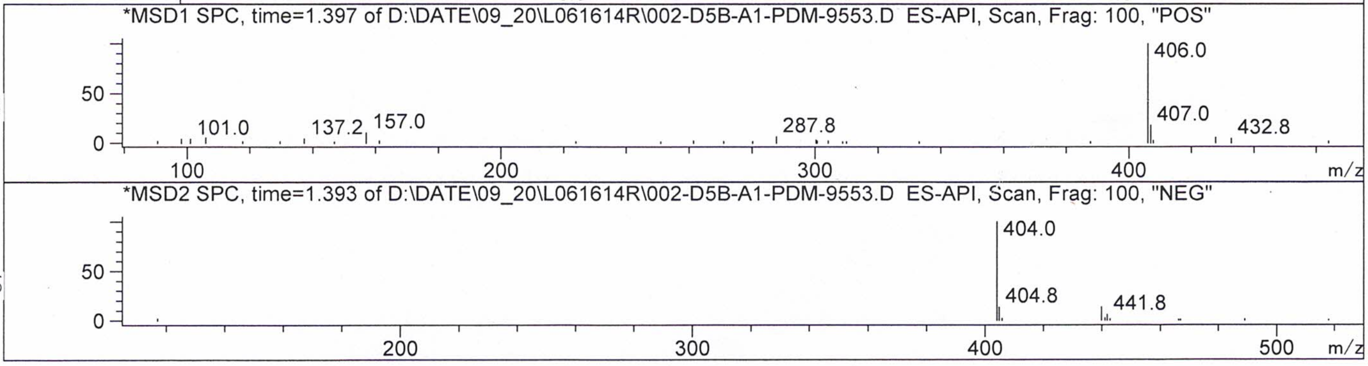

c1

pdm9754.fid

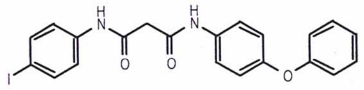

D59

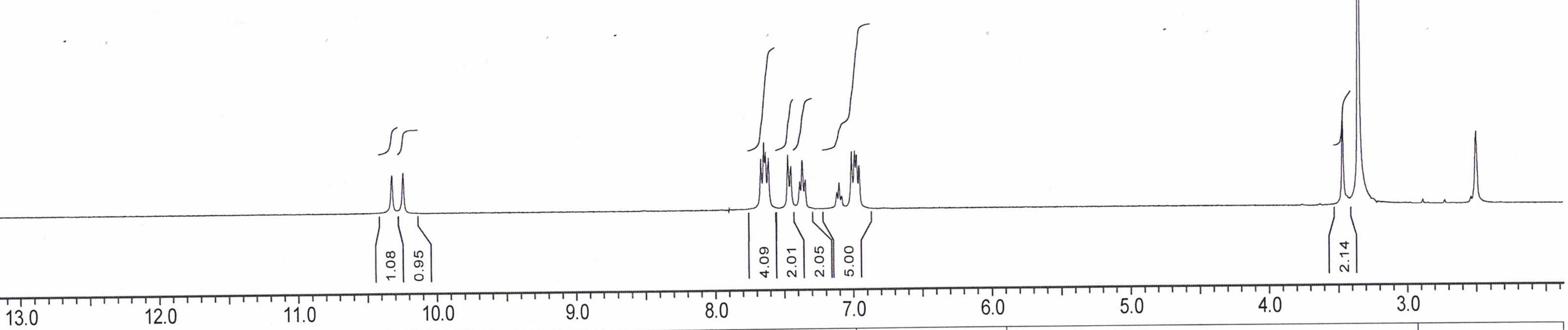

**File name: pdm9754.fid**	Operator:	SF: 399.9831 MHz	NSC: 0	PW: 7.50 usec, RG: 30	SI: 16384
Date: 26-Sep-2017	Solvent: dmso_d6	SW: 7639 Hz	TE: 293 K	AQ: 1.05 sec, RD: 0.00 sec	

c2

MaxPeak: 100.00%
Ret_Time: 1.579 min

Mol Wt 0
Exact Mass

Time	Area%
1.579	100.00

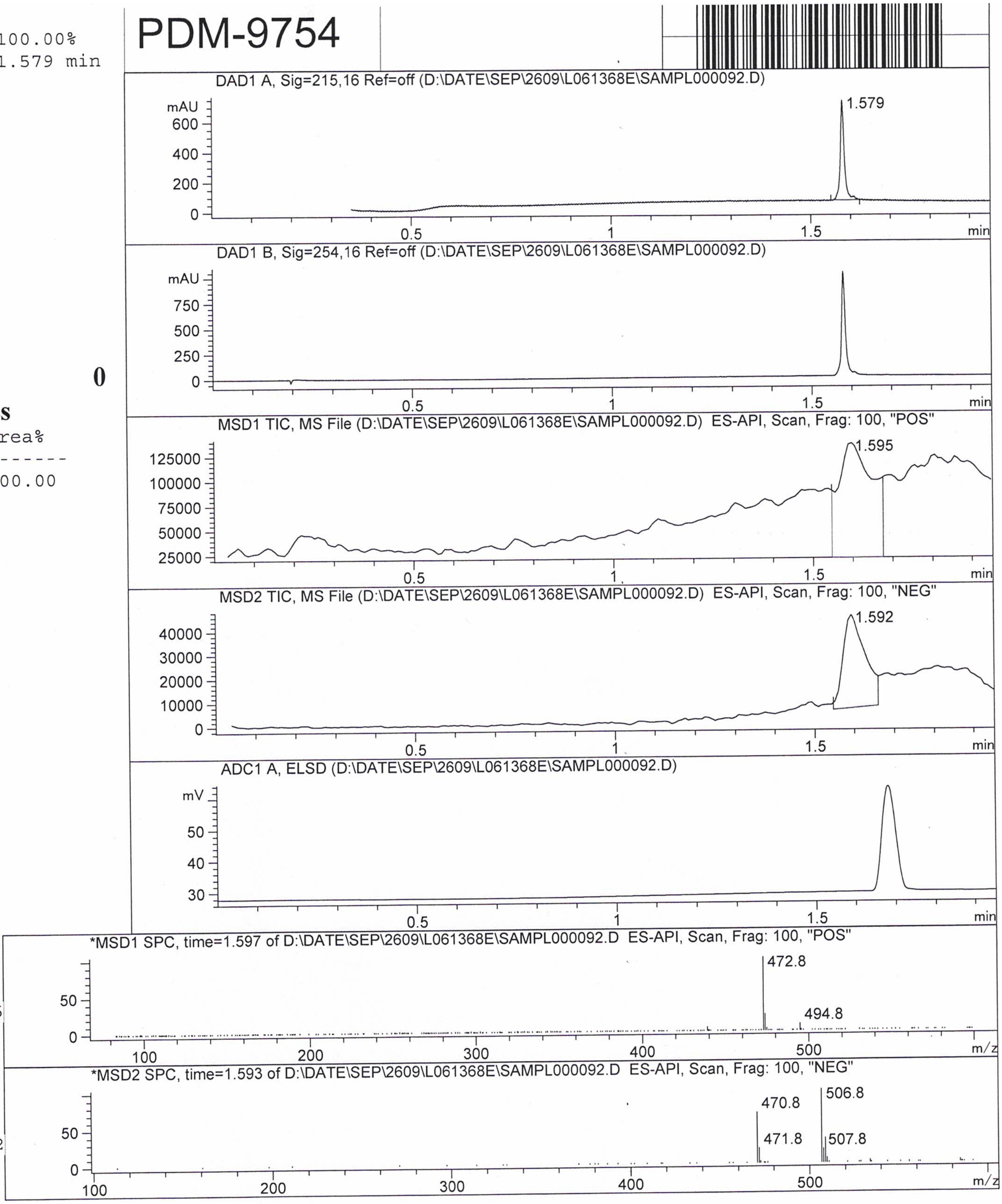

d1

pdm9756.fid

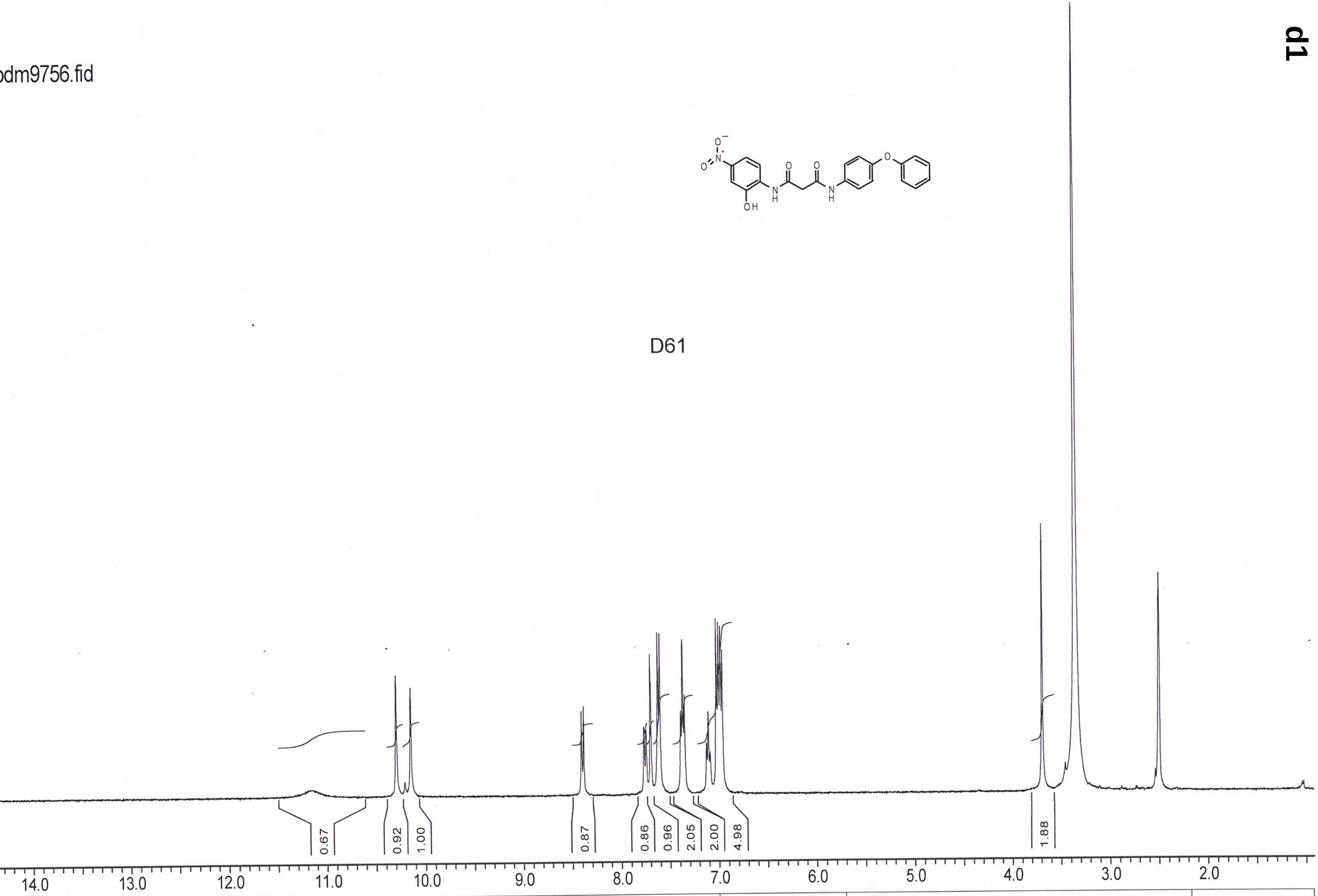

**File name: pdm9756.fid**	Operator:	SF: 399.9831 MHz	NSC: 0	PW: 7.50 usec, RG: 30	SI: 16384
Date: 26-Sep-2017	Solvent: dmso_d6	SW: 7637 Hz	TE: 293 K	AQ: 1.05 sec, RD: 0.00 sec	

d2

MaxPeak: 95.01%
Ret_Time: 1.451 min

PDM-9756

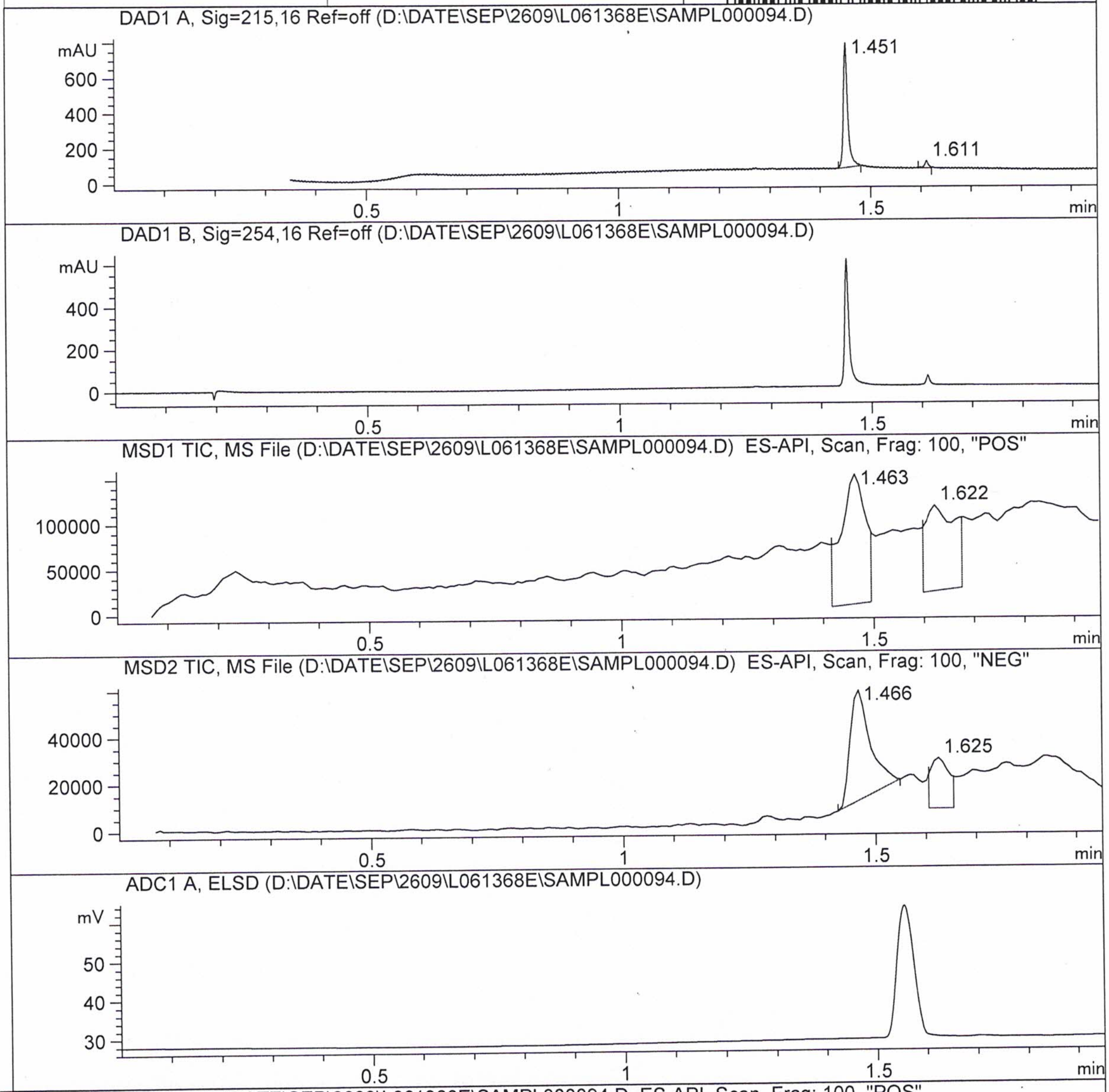

Mol Wt 0

Exact Mass

Time	Area%
1.451	95.01
1.611	4.99

RT 1.463

RT 1.622

RT 1.466

RT 1.625

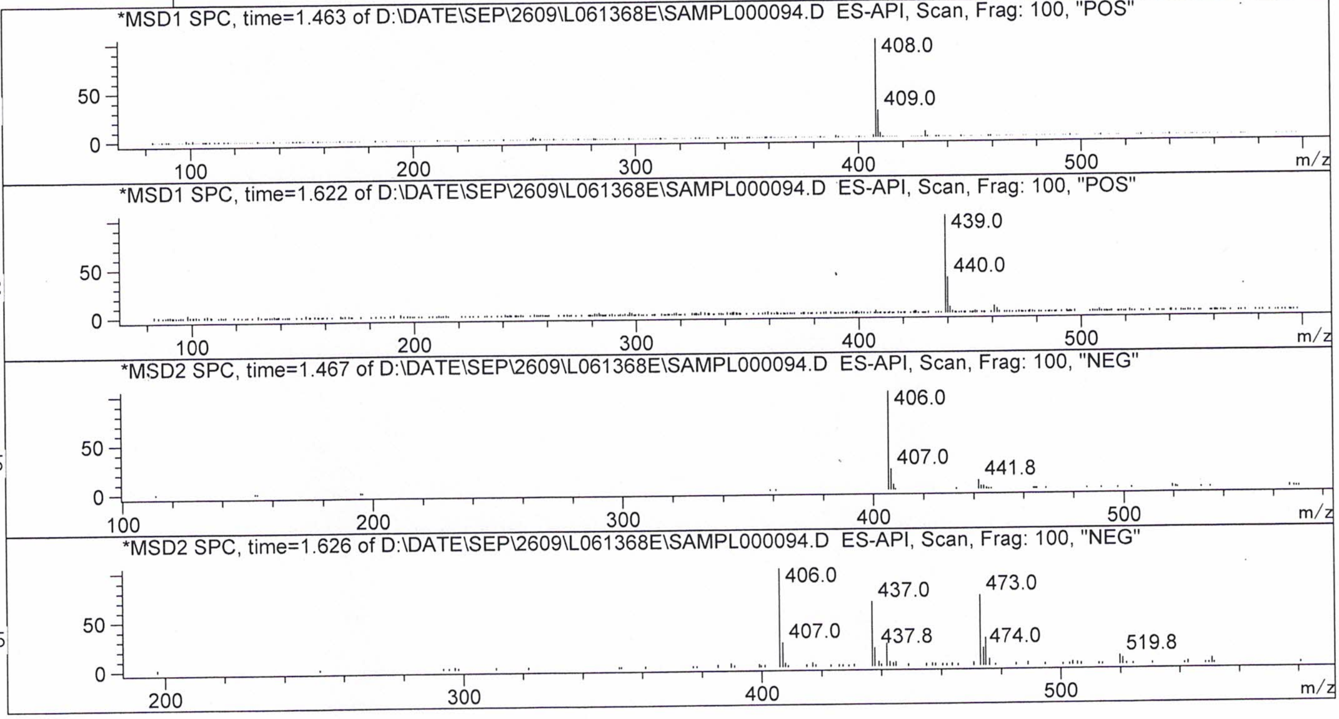

e1

P1LVUM~Y.FID

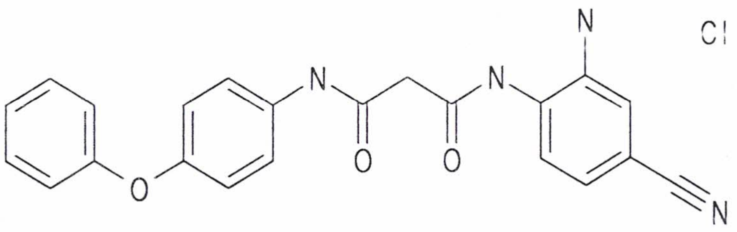

D62

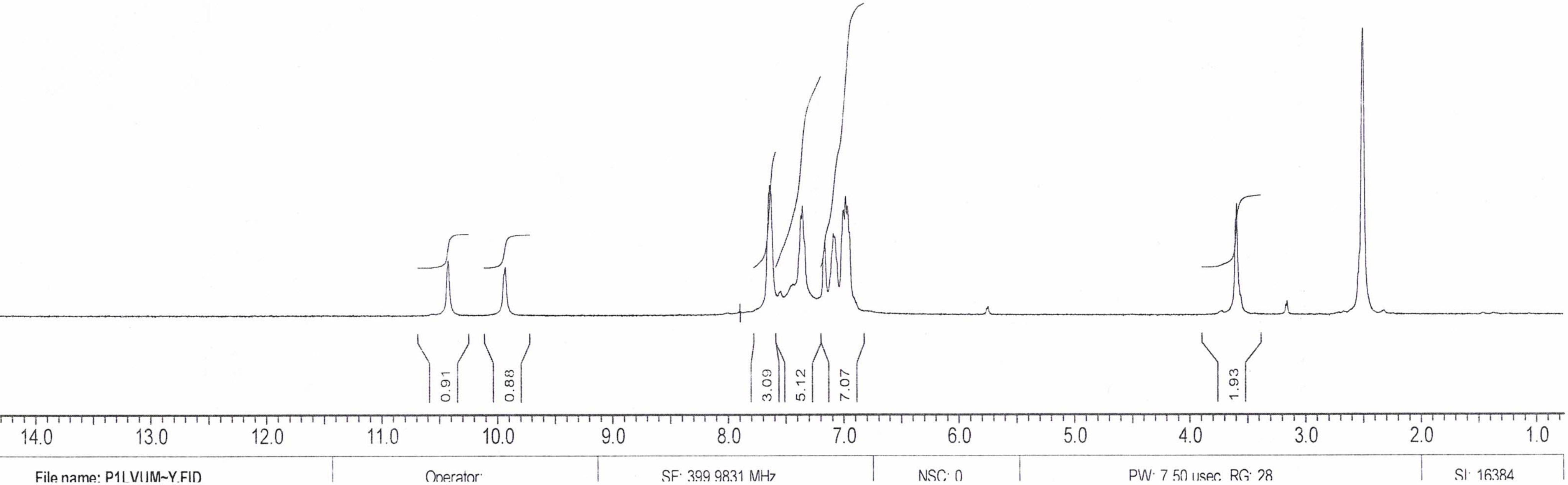

e2

MaxPeak: 96.45%
Ret_Time: 1.364 min

PDM-9847

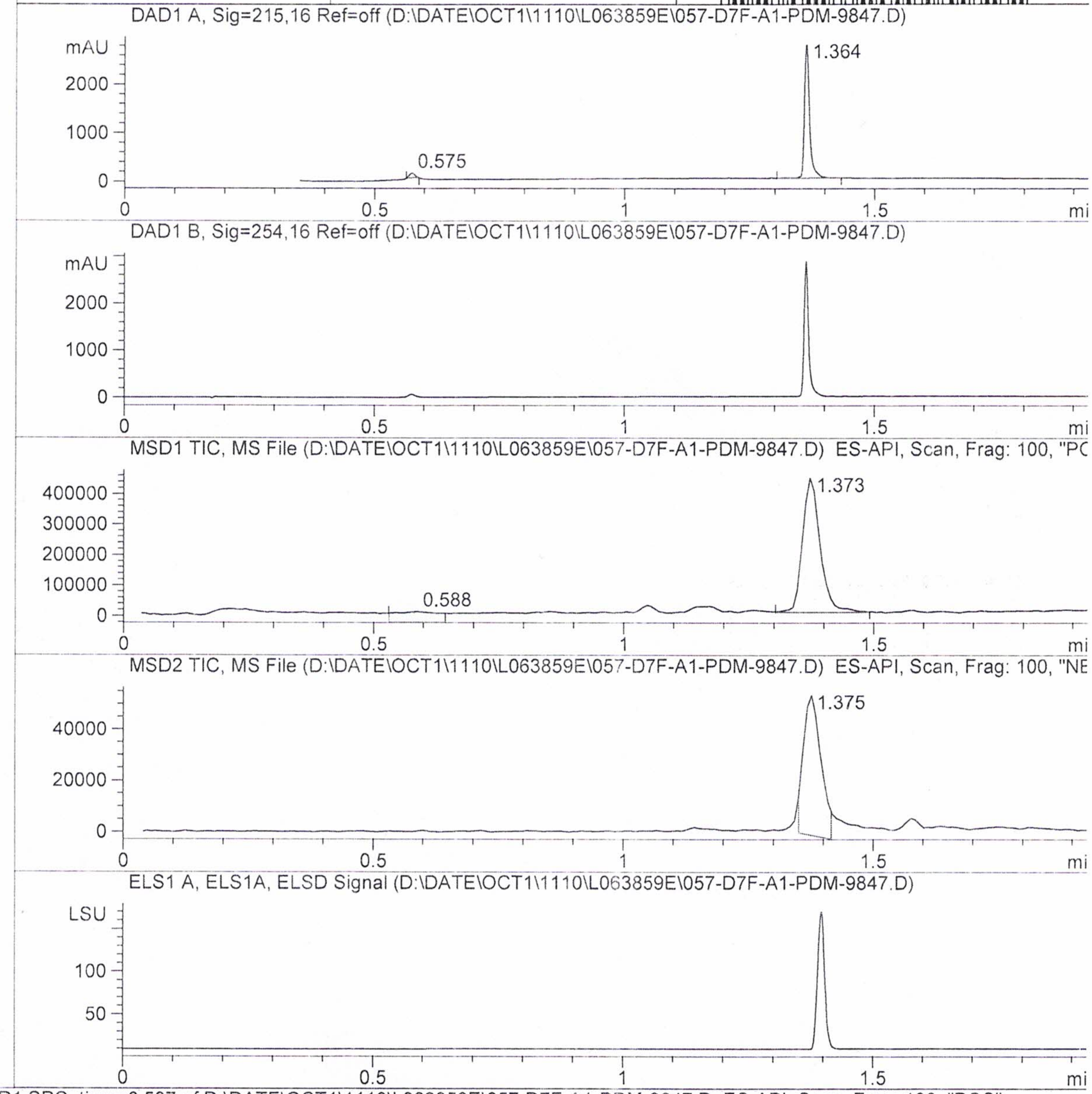

Mol Wt 0

Exact Mass

Time	Area%
0.575	3.55
1.364	96.45

RT 0.588

RT 1.373

RT 1.375

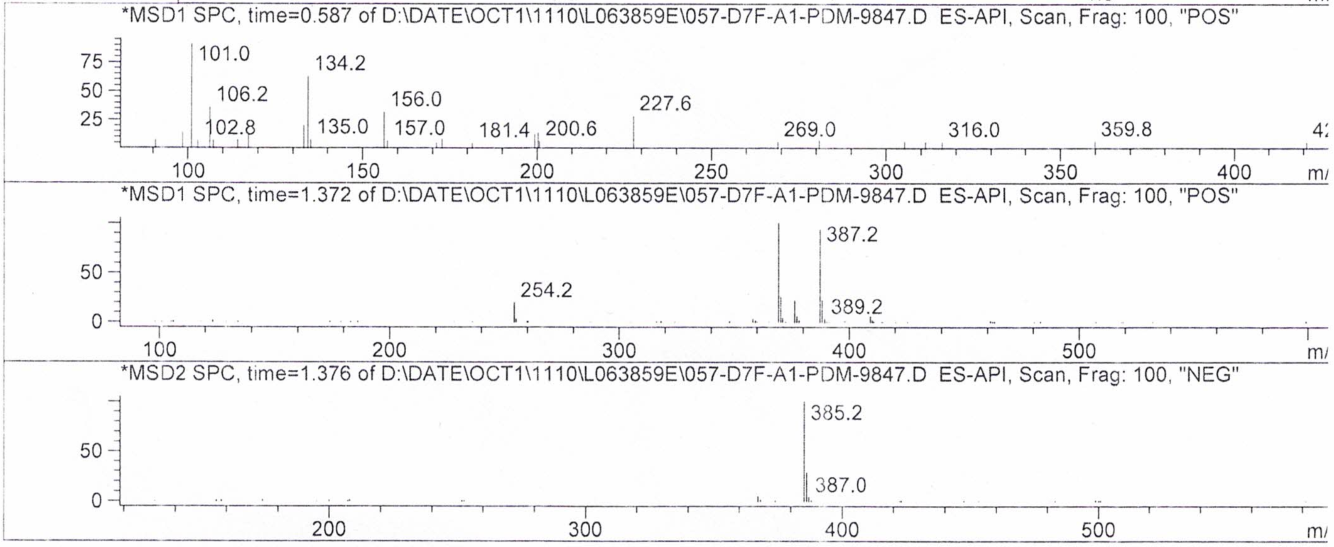

f1

pdm-9805

D63

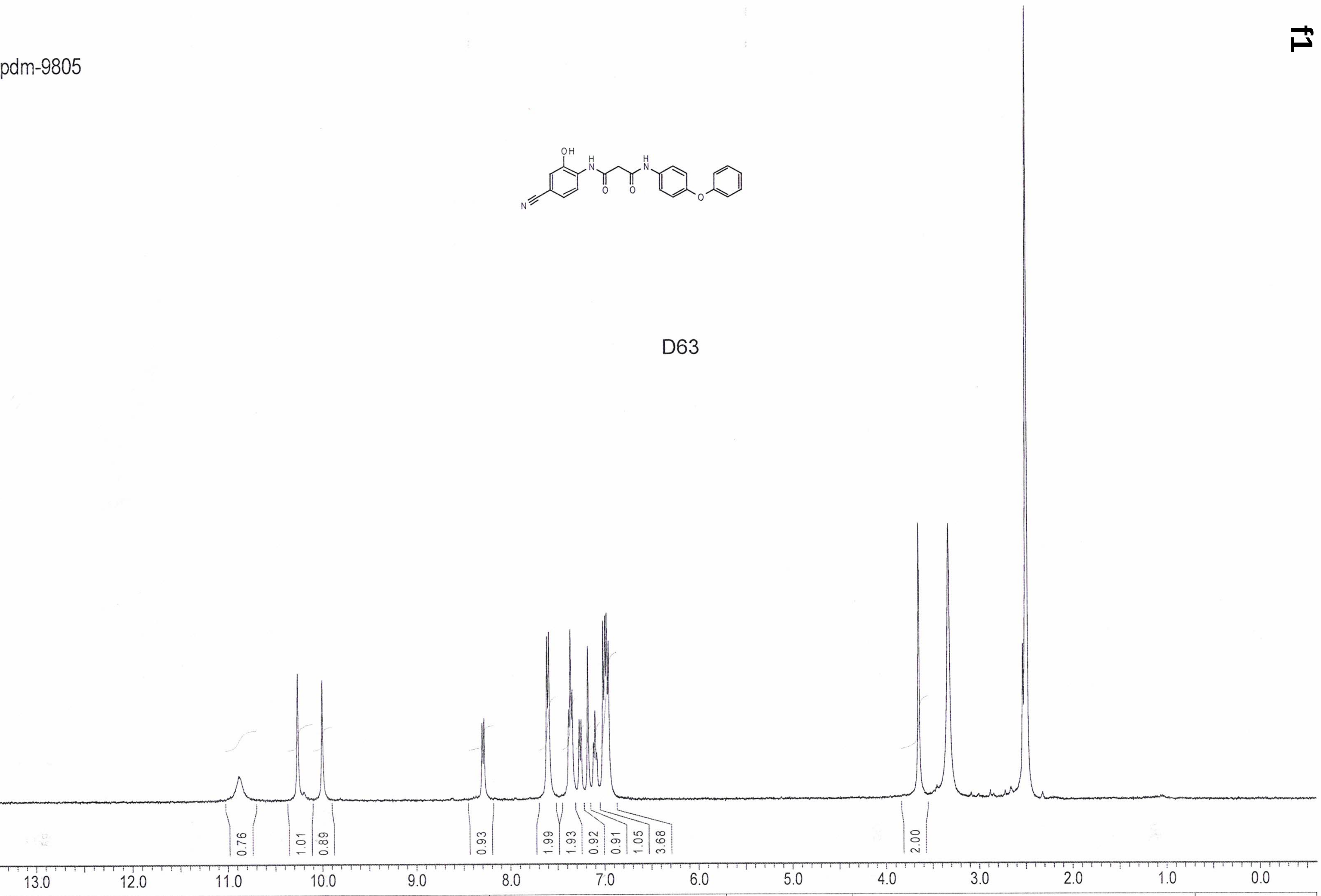

File name: pdm-9805	Operator: USER 1	SF: 400.1300 MHz	NSC: 1	PW: 0.00 usec, RG: 25	SI: 32768
Date: 29-Sep-2017	Solvent: DMSO	SW: 8224 Hz	TE: 300 K	AQ: 1.99 sec, RD: 0.00 sec	

f2

D63

MaxPeak: 100.00%
Ret_Time: 1.394 min

PDM-9805

Mol Wt 0

Exact Mass

#	Time	Area%
1	1.394	100.00

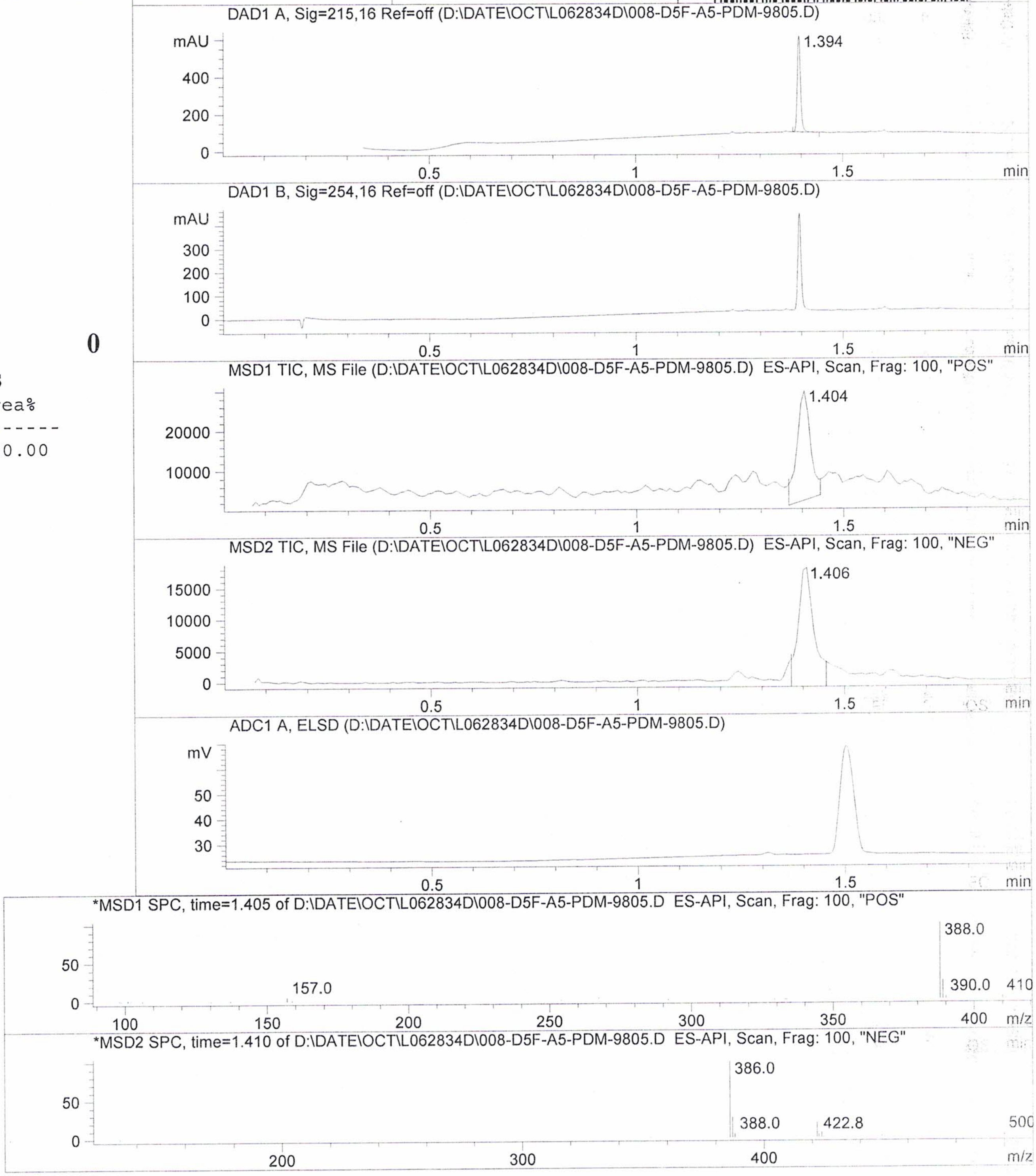

RT 1.404

RT 1.406

g1

pdm9757.fid

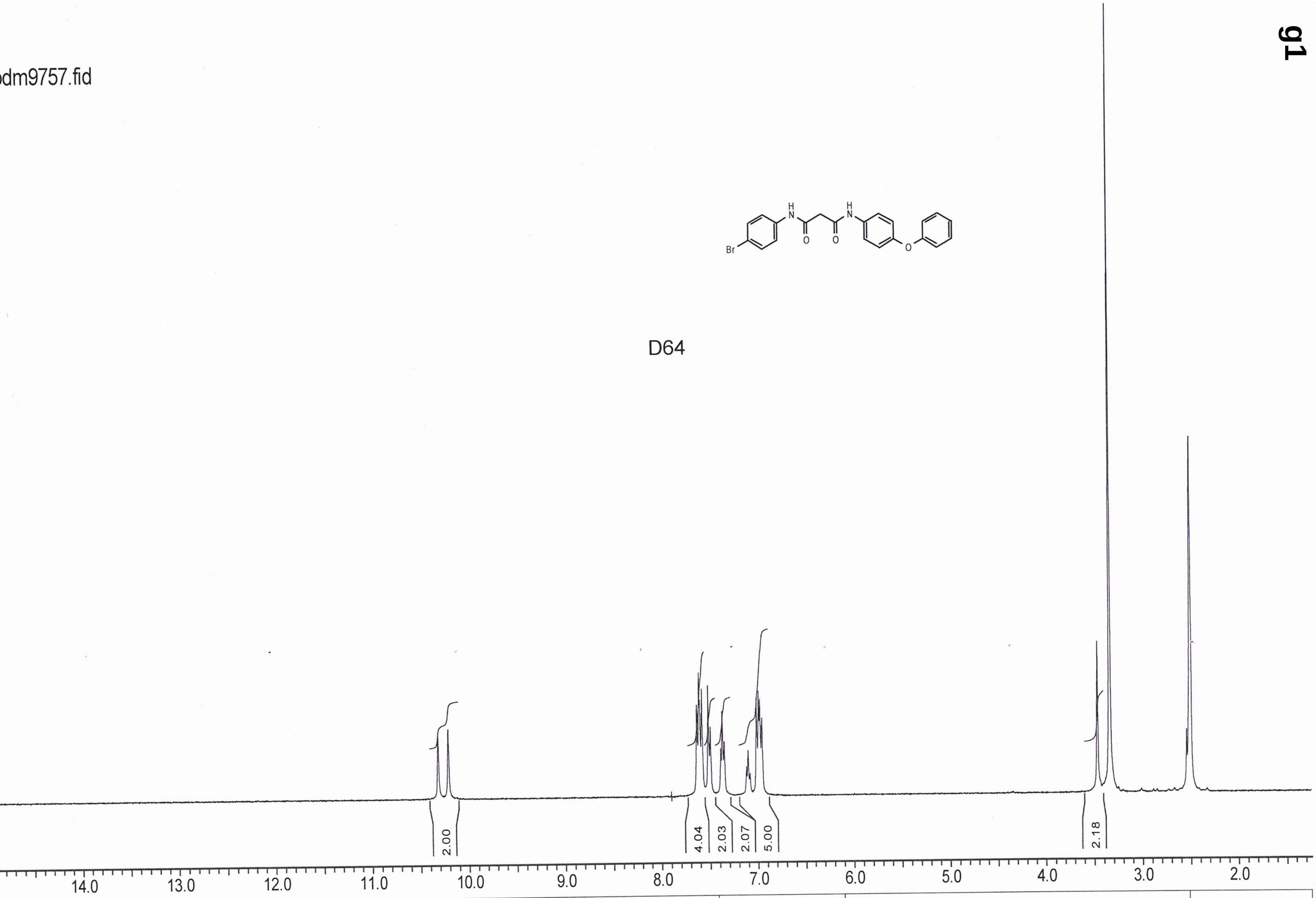

File name: pdm9757.fid	Operator:	SF: 399.9831 MHz	NSC: 0	PW: 7.50 usec, RG: 30	SI: 16384
Date: 26-Sep-2017	Solvent: dmso_d6	SW: 7639 Hz	TE: 293 K	AQ: 1.05 sec, RD: 0.00 sec	

g2

MaxPeak: 97.80%
Ret_Time: 1.558 min

PDM-9757

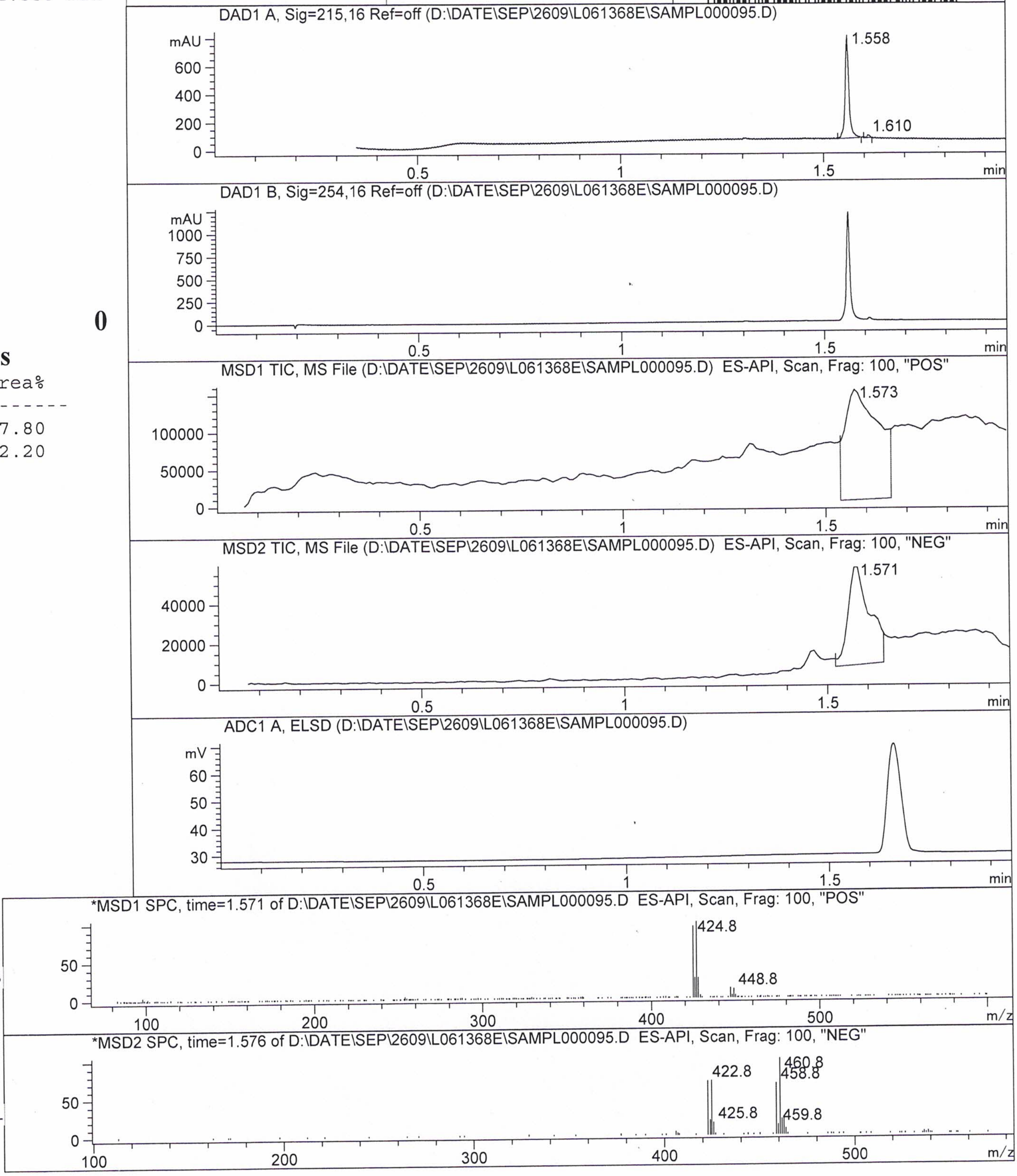

Mol Wt 0

Exact Mass

Time	Area%
1.558	97.80
1.610	2.20

RT 1.573

RT 1.571

h1

^{1}H NMR spectrum of **D88**:

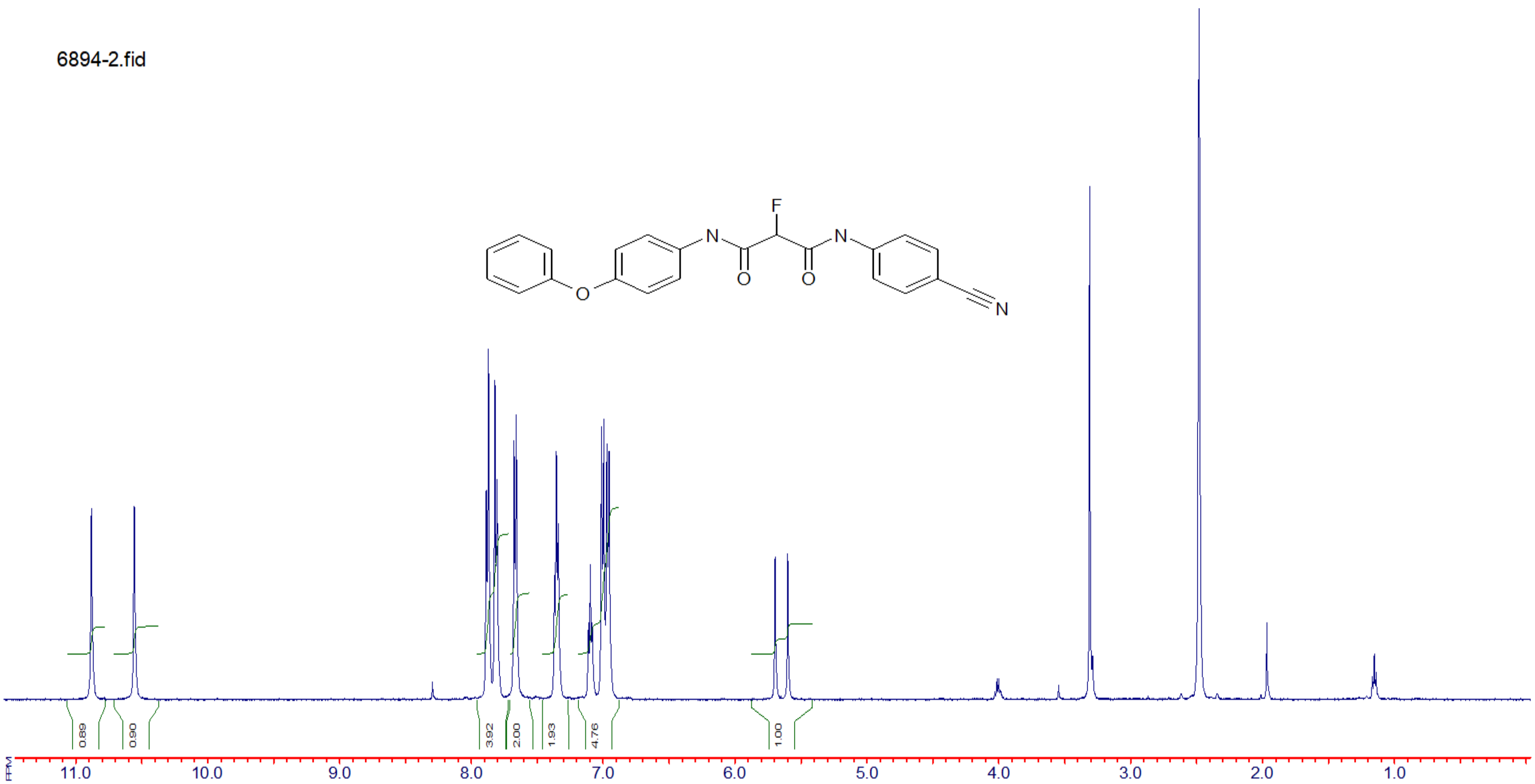

**File name: 6894-2.fid**	Operator:	SF: 499.8203 MHz	NSC: 0	PW: 7.20 usec, RG: 12	SI: 32768
Date: 06-Apr-2020	Solvent: dmso	SW: 9470 Hz	TE: 293 K	AQ: 1.69 sec, RD: 0.00 sec	

h2

LC/MS of **D88**:

MaxPeak: 100.00%
Ret_Time: 1.381 min

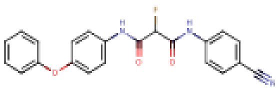

Mol Wt 389.38
Exact Mass 389.13

#	Time	Area%
1	1.381	100.00

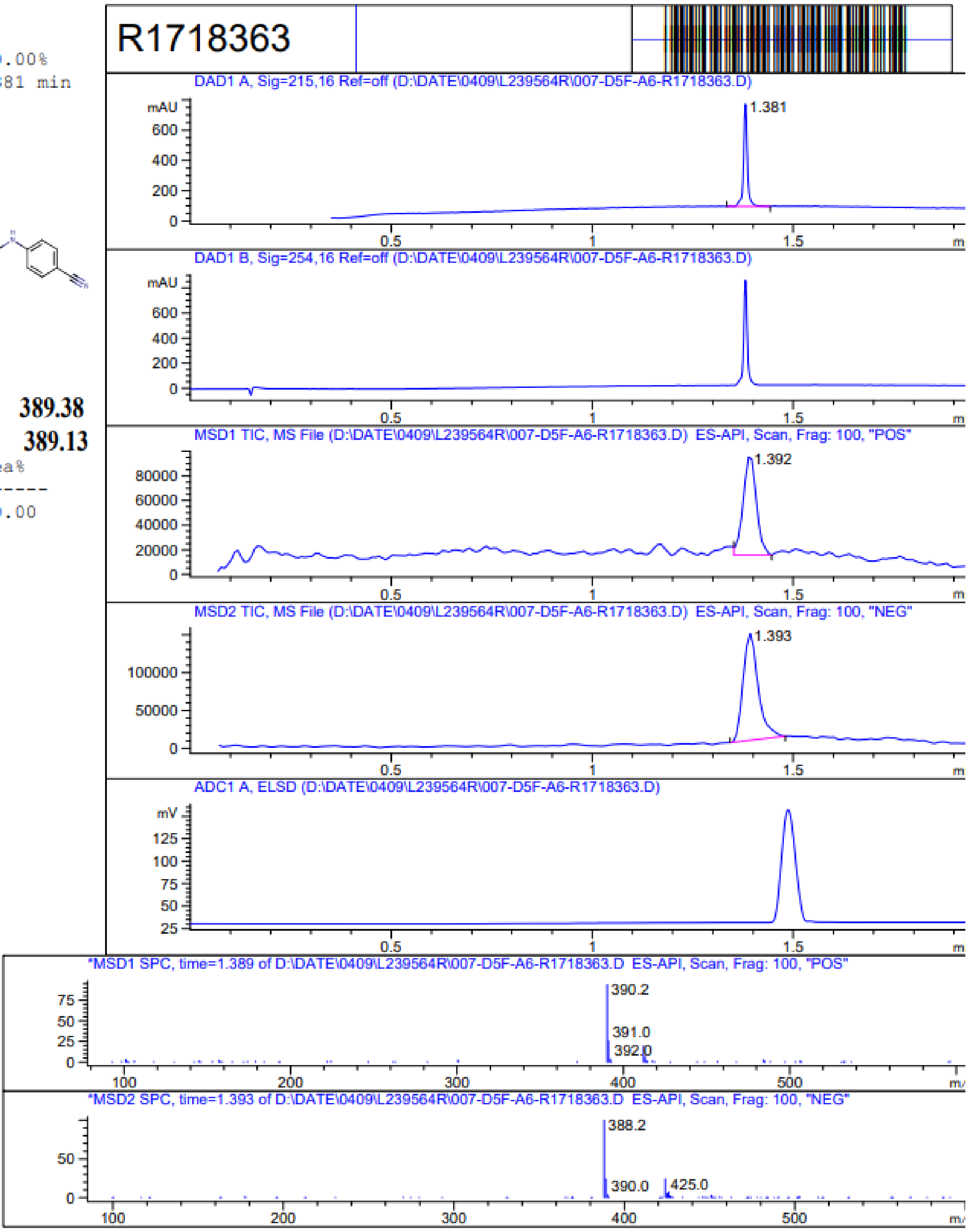

RT 1.392

RT 1.393
