# Supplementary material for: Tackling recalcitrant Pseudomonas aeruginosa infections in critical illness via anti-virulence monotherapy
Source: Nat Commun. 2022 Aug 30;13:5103. doi: 10.1038/s41467-022-32833-9 (PMC9428149; doi:10.1038/s41467-022-32833-9)
Supplement: Supplementary file 3 — Reporting Summary [file 41467_2022_32833_MOESM3_ESM.pdf]

nature portfolio

Corresponding author(s): Laurence G. Rahme

Last updated by author(s): 06/02/2022

Reporting Summary

Nature Portfolio wishes to improve the reproducibility of the work that we publish. This form provides structure for consistency and transparency in reporting. For further information on Nature Portfolio policies, see our Editorial Policies and the Editorial Policy Checklist.

Statistics

For all statistical analyses, confirm that the following items are present in the figure legend, table legend, main text, or Methods section.

n/a	Confirmed	
☐	☒	The exact sample size (*n*) for each experimental group/condition, given as a discrete number and unit of measurement
☐	☒	A statement on whether measurements were taken from distinct samples or whether the same sample was measured repeatedly
☐	☒	The statistical test(s) used AND whether they are one- or two-sided<br>*Only common tests should be described solely by name; describe more complex techniques in the Methods section.*
☒	☐	A description of all covariates tested
☒	☐	A description of any assumptions or corrections, such as tests of normality and adjustment for multiple comparisons
☐	☒	A full description of the statistical parameters including central tendency (e.g. means) or other basic estimates (e.g. regression coefficient) AND variation (e.g. standard deviation) or associated estimates of uncertainty (e.g. confidence intervals)
☐	☒	For null hypothesis testing, the test statistic (e.g. *F*, *t*, *r*) with confidence intervals, effect sizes, degrees of freedom and *P* value noted<br>*Give P values as exact values whenever suitable.*
☒	☐	For Bayesian analysis, information on the choice of priors and Markov chain Monte Carlo settings
☒	☐	For hierarchical and complex designs, identification of the appropriate level for tests and full reporting of outcomes
☒	☐	Estimates of effect sizes (e.g. Cohen's *d*, Pearson's *r*), indicating how they were calculated

Our web collection on statistics for biologists contains articles on many of the points above.

Software and code

Policy information about availability of computer code

Data collection	no computer code used- Standard operating system software was used for data acquisition for confocal images, Microplate reader and RT-PCR.
Data analysis	NIS-Elements AR 5.21.03, NIH image J; QuantStudio; Magellan; i-Control; Biacore T200 2.0; GraphPad Prism 9.2.0;

For manuscripts utilizing custom algorithms or software that are central to the research but not yet described in published literature, software must be made available to editors and reviewers. We strongly encourage code deposition in a community repository (e.g. GitHub). See the Nature Portfolio guidelines for submitting code & software for further information.

Data

Policy information about availability of data

All manuscripts must include a data availability statement. This statement should provide the following information, where applicable:

- Accession codes, unique identifiers, or web links for publicly available datasets
- A description of any restrictions on data availability
- For clinical datasets or third party data, please ensure that the statement adheres to our policy

The authors declare that data supporting the findings of this study are included in the paper and supporting information except Crystal structure of MvfR ligand binding domain in complex with M64 which is available at DOI: 10.2210/pdb6b8a/pdb

Human research participants

Policy information about studies involving human research participants and Sex and Gender in Research.

Reporting on sex and gender	N/A
Population characteristics	N/A
Recruitment	N/A
Ethics oversight	N/A

Note that full information on the approval of the study protocol must also be provided in the manuscript.

Field-specific reporting

Please select the one below that is the best fit for your research. If you are not sure, read the appropriate sections before making your selection.

☒ Life sciences ☐ Behavioural & social sciences ☐ Ecological, evolutionary & environmental sciences

For a reference copy of the document with all sections, see nature.com/documents/nr-reporting-summary-flat.pdf

Life sciences study design

All studies must disclose on these points even when the disclosure is negative.

Sample size	All sample size are described in figure legends. Sample size were decided based on the estimate from previous similar studies (Starkey, M. et al. 2014.PLoS pathogens 10, e1004321, doi:10.1371/journal.ppat.1004321; Adiliaghdam, F. et al. 2019. Mol Med Rep 19, 4057-4066, doi:10.3892/mmr.2019.10071; Schutz, C. et al. 2021. Adv Sci (Weinh) 8, e2004369, doi:10.1002/advs.202004369). Minimum 3 biological replicates were used. All experiments were performed at least 3 times unless otherwise mentioned. For in vivo studies- at least 5 animals per group were used and experiments were repeated at least twice.
Data exclusions	No data were excluded
Replication	Experiments were repeated at least twice yielding similar data. Numbers of replicates are reported in the manuscript.
Randomization	For in vivo experiments age-matched mice were randomized into treatment groups. For in vitro experiments all samples were allocated randomly in cultures(control and treatment).
Blinding	Blinding was not done in any of the experiment. For in vitro studies experiment, data accusation and analysis were done by same researcher. For in vivo studies researchers were not blinded since certain animal groups received repetitive treatment over the course of the study and same researcher performed the experiment, data accusation and analysis.

Reporting for specific materials, systems and methods

We require information from authors about some types of materials, experimental systems and methods used in many studies. Here, indicate whether each material, system or method listed is relevant to your study. If you are not sure if a list item applies to your research, read the appropriate section before selecting a response.

Materials & experimental systems

n/a	Involved in the study	
☐	☒	Antibodies
☐	☒	Eukaryotic cell lines
☒	☐	Palaeontology and archaeology
☐	☒	Animals and other organisms
☒	☐	Clinical data
☒	☐	Dual use research of concern

Methods

n/a	Involved in the study	
☒	☐	ChIP-seq
☒	☐	Flow cytometry
☒	☐	MRI-based neuroimaging

Antibodies

Antibodies used	Primary antibody rabbit polyclonal anti-claudin-1, Catalog#71-7800, Invitrogen<br>Secondary antibody goat anti-rabbit, Catalog#ab150061; Abcam
Validation	Claudin-1: https://www.thermofisher.com/antibody/product/Claudin-1-Antibody-clone-MH25-Polyclonal/71-7800<br>Secondary antibody goat anti-rabbit: https://www.abcam.com/donkey-rabbit-igg-hl-alexa-fluor-488-preadsorbed-ab150061.html

Eukaryotic cell lines

Policy information about cell lines and Sex and Gender in Research

Cell line source(s)	Four different cell lines were used, namely RAW 264.7 (macrophage), Caco-2 (colon epithelial cells), Hep G2 (liver cells), and A549 (lung epithelial cells) were obtained from ATCC.
Authentication	All cell lines used were commercially available and purchased from ATCC. Nevertheless, prior to use were checked for morphology, and cell proliferation.
Mycoplasma contamination	All cell lines were negative for mycoplasma. We performed periodic assays to detect mycoplasma with PlasmoTest™ kit (Invivogen). In addition, we also used Hoechst 33258, to reveal potential mycoplasma infection.
Commonly misidentified lines<br>(See ICLAC register)	No misidentified cell lines were used.

Animals and other research organisms

Policy information about studies involving animals; ARRIVE guidelines recommended for reporting animal research, and Sex and Gender in Research

Laboratory animals	C57BL/6J 10 week old Male and CD-1 6 week old male mice were used.
Wild animals	No wild animals were used in the study.
Reporting on sex	No sex-based study was performed.
Field-collected samples	No field collected samples were used in the study.
Ethics oversight	Animal protocols were reviewed and approved by the Institutional Animal Care and Use Committee (IACUC) at the MGH (protocol no. 2006N000093) and are in strict accordance with the guidelines of the Committee on Animals of the MGH, Harvard Medical School (Boston, USA), and the regulations of the Subcommittee on Research Animal Care of the MGH and the National Institutes of Health.<br><br>All experiments involving animals for PK studies were carried out by Aptuit (Verona) S.rl, an Evotec Company, Italy in accordance with the European directive 2010/63/UE governing animal welfare and protection, which is acknowledged by the Italian Legislative Decree no 26/2014 and according to the company policy on the care and use of laboratories animals. All the studies were revised by the Animal Welfare Body and approved by Italian Ministry of Health (authorization n. -PR)

Note that full information on the approval of the study protocol must also be provided in the manuscript.
